# Alcohol‐Processable All‐Polymer n‐Type Thermoelectrics

**DOI:** 10.1002/advs.202401952

**Published:** 2024-04-22

**Authors:** Xinyi Fan, Jian Liu, Xiaozheng Duan, Hongxiang Li, Sihui Deng, Yazhuo Kuang, Jingyu Li, Chengjiang Lin, Bin Meng, Junli Hu, Shumeng Wang, Jun Liu, Lixiang Wang

**Affiliations:** ^1^ State Key Laboratory of Polymer Physics and Chemistry Changchun Institute of Applied Chemistry Chinese Academy of Sciences Changchun Jilin 130022 P. R. China; ^2^ School of Applied Chemistry and Engineering University of Science and Technology of China Hefei Anhui 230026 P. R. China; ^3^ College of Polymer Science and Engineering State Key Laboratory of Polymer Materials Engineering Sichuan University Chengdu Sichuan 610065 P. R. China; ^4^ Key Laboratory of UV‐Emitting Materials and Technology (Northeast Normal University) Ministry of Education Changchun Jilin 130024 P. R. China

**Keywords:** all‐polymer n‐type thermoelectrics, conducting materials, n‐doping, organic thermoelectrics, polymer dopant

## Abstract

The general strategy for n‐type organic thermoelectric is to blend n‐type conjugated polymer hosts with small molecule dopants. In this work, all‐polymer n‐type thermoelectric is reported by dissolving a novel n‐type conjugated polymer and a polymer dopant, poly(ethyleneimine) (PEI), in alcohol solution, followed by spin‐coating to give polymer host/polymer dopant blend film. To this end, an alcohol‐soluble n‐type conjugated polymer is developed by attaching polar and branched oligo (ethylene glycol) (OEG) side chains to a cyano‐substituted poly(thiophene‐alt‐co‐thiazole) main chain. The main chain results in the n‐type property and the OEG side chain leads to the solubility in hexafluorineisopropanol (HFIP). In the polymer host/polymer dopant blend film, the Coulombic interaction between the dopant counterions and the negatively charged polymer chains is reduced and the ordered stacking of the polymer host is preserved. As a result, the polymer host/polymer dopant blend exhibits the power factor of 36.9 µW m^−1^ K^−1^, which is one time higher than that of the control polymer host/small molecule dopant blend. Moreover, the polymer host/polymer dopant blend shows much better thermal stability than the control polymer host/small molecule dopant blend. This research demonstrates the high performance and excellent stability of all‐polymer n‐type thermoelectric.

## Graphical abstract

A high‐performance n‐type thermoelectric is developed by dissolving a new polymer host and a polymer dopant poly(ethyleneimine) (PEI) in hexafluoroisopropanol. The new combination reduces Coulombic interaction between the dopant counterions and the charged polymer chains, which leads to preserved ordered stacking of the polymer host. As a result, the Seebeck coefficient is enhanced and consequently higher power factor and improved thermal stability.

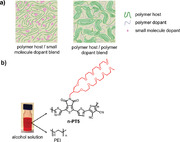

## 1 Introduction

**Scheme 1:**
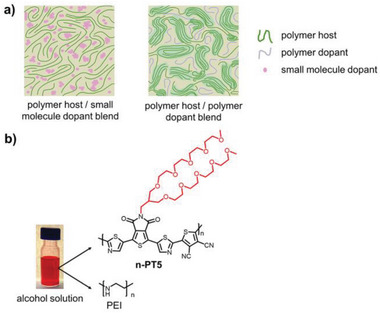
a) Illustration of the aggregation status of the polymer host and the distribution of small molecular dopants and polymer dopants in film. b) Photograph of the n‐PT5: PEI blend alcohol solution (the solvent is hexafluoroisopropanol) and chemical structures of n‐PT5 and PEI.

Organic thermoelectric (OTE) devices convert heat into electricity, or vice versa, which enables a wide range of applications in wearable electronics, micro‐cooling machinery, and bioelectronics.^[^ ^1^ ^]^ For high‐performance thermogenerators, both efficient n‐type and p‐type thermoelectric materials with comparable performance are required.^[^ ^2^ ^]^ While most research focuses on p‐type polymer thermoelectric materials, n‐type polymer thermoelectric materials are far less developed.^[^ ^1^, ^3^ ^]^ Doping of organic semiconductors is always necessary for high‐performance OTE because doping leads to filled charge carrier traps and increased charge carrier concentration.^[^ ^4^ ^]^ Recently, a variety of n‐dopants have been employed in OTEs, such as 1,3‐dimethyl‐2‐phenylbenzimidazoline (*N*‐DMBI),^[^ ^6^ ^]^ tetrakis(dimethylamino)ethylene (TDAE),^[^ ^7^ ^]^ bis(cyclopentadienyl)cobalt (CoCp_2_),^[^ ^8^ ^]^ N‐heterocyclic carbenes (NHCs),^[^ ^9^ ^]^ triaminomethane derivatives (TAMs),^[^ ^10^ ^]^ etc.^[^ ^11^ ^]^ A large family of n‐type conjugated polymers has also been developed ^[^ ^5^ ^]^ and several new doping methods have also been reported.^[^ ^12^, ^13^ ^]^ These efforts have resulted in dramatic enhancement of n‐type thermoelectric performance of conjugated polymers in recent several years.

n‐Dopants are always organic small molecules.^[^ ^14^ ^]^ The common doping method is to blend polymer hosts and small molecule dopants in solution to obtain polymer/small molecule blend film by spin‐coating.^[^ ^8^, ^15^ ^]^ However, small molecule dopants tend to self‐aggregate during the film‐forming process,^[^ ^16^ ^]^ resulting in low doping efficiency and high optimal dopant concentration. High loading of small molecule dopants severely destroys the ordered packing of polymer hosts (see **Scheme** **1a**), leading to degraded thermoelectric performance. Moreover, polymer host/small‐molecule dopant blends suffer from unsatisfactory thermal stability due to the aggregation and sublimation of small‐molecule dopants.^[^ ^17^ ^]^ A possible strategy to circumvent these problems of polymer host/small molecule dopant blends is to use polymer host/polymer dopant,^[^ ^18^ ^]^ i.e., all‐polymer n‐type thermoelectrics. Polymer/polymer blends always exhibit not only excellent morphology stability but also unique phase separation behavior for enhanced thermoelectric performance. Generally, n‐type conjugated polymers bear alkyl side chains and are soluble in nonpolar solvents, while the available polymer n‐dopant, poly(ethyleneimine) (PEI), is only soluble in polar solvents. It is difficult to mix them in a solution. Therefore, there are very few reports on n‐type thermoelectrics of polymer host/polymer dopant blends.^[^ ^19^, ^20^ ^]^ To overcome the solubility issue, scientists have tried to either disperse n‐type conjugated polymers in polar solvent or develop non‐polar polymer dopants. The resulting polymer host/polymer dopant blend films achieve excellent stability or thermoelectric performance.

In this work, for the first time, we develop an alcohol‐soluble n‐type conjugated polymer (n‐PT5, see Scheme 1b) for thermoelectric application by attaching polar and branched oligo(ethylene glycol) (OEG) side chains to a cyano‐substituted poly(thiophene‐alt‐co‐thiazole) main chain. The main chain results in the n‐type property and the OEG side chain leads to the alcohol solubility. All‐polymer n‐type thermoelectric is demonstrated by dissolving the n‐type conjugated polymer and a polymer dopant in an alcohol solution, followed by spin‐coating to give a polymer host/polymer dopant blend film. In the film, the Coulombic interaction between the dopant counterions and the negatively charged polymer chains is reduced and the ordered stacking of the polymer host is preserved, leading to excellent n‐type thermoelectric performance and improved thermal stability. This research provides feasible guidelines for developing new types of n‐type conjugated polymers for high‐performance and stable n‐type organic thermoelectrics.

## 2 Results and Discussion

Scheme 1b shows the chemical structure of the alcohol‐soluble n‐type conjugated polymer, n‐PT5. The backbone of n‐PT5 consists of alternating thiazole‐flanked thieno[3,4‐*c*]pyrrole‐4,6‐dione (TPD) unit and 3,4‐dicyanothiophene (2CN‐T) unit. Cyano and imide groups with strong electron‐pulling ability are introduced to the polymer backbone of n‐PT5 for low‐lying LOMO energy level and improved n‐doping efficiency. In addition, the insertion of a thiazole unit in the polymer backbone leads to the coplanar‐optimized conformation of the polymer backbone. This is supported by the optimized conformation of four repeating units of the polymer backbone of n‐PT5 (see Figure S1, Supporting Information), which is obtained by density‐functional theory (DFT) calculations at the B3LYP/6‐31G (d, p) level. The coplanar optimized conformation of the polymer backbone is expected to improve close π‐stacking of the polymer backbones in thin film and facilitate electron transport.^[^ ^21^ ^]^ Long and branched oligo(ethylene glycol) side chains are connected to the TPD unit to improve the solubility of the polymer in alcohol. In a previous study conducted by Liu et al., it was discovered that the polar side chains are effective in enhancing the miscibility of the host and dopant, which subsequently leads to improved doping efficiency.^[^ ^22^ ^]^

**Scheme 2:**
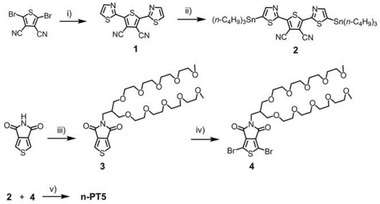
Synthetic routes of n‐PT5. (Reagents and conditions: i) 2‐(tri‐*n*‐butylstannyl)thiazole, Pd(PPh_3_)_4_, toluene, 115 °C. ii) LDA, Bu_3_SnCl, THF, −78 °C. iii) K_2_CO_3_, DMF, r.t. iv) NBS, H_2_SO_4_, CF_3_COOH, r.t., v) Pd_2_(dba)_3_, P(*o*‐tolyl)_3_, toluene, 115 °C.).

The synthetic routes of n‐PT5 are illustrated in **Scheme** **2** and the synthesis methods are provided in the Supporting Information. Stille coupling of 2,5‐dibromo‐3,4‐dicyanothiophene and 2‐(tri‐*n*‐butylstannyl)thiazole afforded the thiazole‐substituted 3,4‐dicyanothiophene, which was further lithiated and reacted with tri‐*n*‐butyltin chloride to afford the di(tributyltin) monomer **2**. The synthesis of tolylated branched oligo(ethylene glycol) is according to previous paper.^[^ ^5b^ ^,^ ^23^ ^]^Substitution of TPD by iodinated branched oligo(ethylene glycol) in the presence of potassium carbonate gave compound **3**. The dibromo monomer **4** is synthesized by bromination of **3** with N‐bromosuccinimide. Stille polycondensation of **2** and **4** afforded n‐PT5. The chemical structure of n‐PT5 is confirmed by ^1^H NMR and elemental analysis. n‐PT5 is readily soluble in not only chlorinated solvents, such as chloroform, chlorobenzene, etc., but also some specific alcohol solvents, such as hexafluoroisopropanol (HFIP). The alcohol solubility of n‐PT5 is due to the polar branched oligo(ethylene glycol) side chains. The molecular weight of n‐PT5 was measured by gel permeation chromatography (GPC) at room temperature using HFIP as the eluent. The number‐average molecular weight (*M*_n_) and polydispersity index (PDI) of n‐PT5 are 98.9 kDa and 1.9, respectively. n‐PT5 exhibits excellent thermal stability with thermal decomposition temperatures (*T*_d_) at a 5% weight loss of 340 °C (Figure S4, Supporting Information).

**Figure 1:**
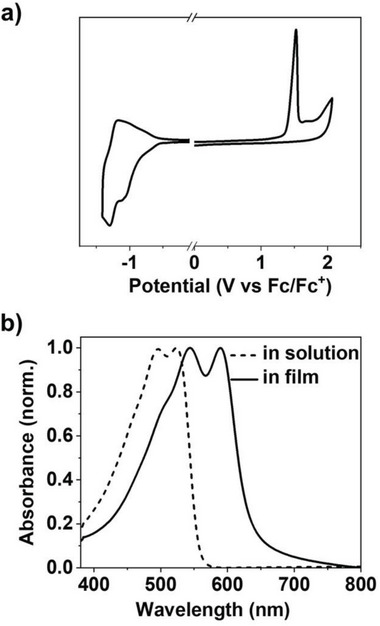
a) Cyclic voltammograms of n‐PT5 in thin film and b) UV–vis–NIR absorption spectra of n‐PT5 in solution and film.

**Figure** **1a** shows the cyclic voltammogram of n‐PT5 in a thin film. Both oxidation and onset reduction waves can be observed. According to the onset oxidation and reduction potentials, the lowest unoccupied molecular orbital (LUMO) energy level and highest occupied molecular orbital (HOMO) energy levels are estimated to be −4.24 and −6.19 eV, respectively. The LUMO and HOMO levels are the lowest reported for polythiophene derivatives to date. This is due to the introduction of electron‐withdrawing cyano groups and imide groups in the polymer backbone as well as the insertion of thiazole unit in the polymer backbone. The low‐lying LUMO level is expected to facilitate charge/hydride transfer between the polymer and n‐dopants, which may thermodynamically improve n‐doping efficiency.^[^ ^24^ ^]^ Figure 1b shows the ultraviolet–visible–near infrared (UV–vis–NIR) absorption spectra of n‐PT5 in HFIP solution and in thin film. n‐PT5 shows two absorption peaks at 496 and 524 nm in solution and exhibits two absorption peaks at 545 and 589 nm in thin film. The redshift of absorption spectra from in solution to in thin film of n‐PT5 is ≈60 nm. This is attributed to the more vital intermolecular interaction and the enhanced planarity of the polythiophene backbone in the solid state. n‐PT5 film shows an onset absorption wavelength at 636 nm, corresponding to the optical bandgap E_g_^opt^ of 1.95 eV.

Poly(ethyleneimine) (PEI), which is readily soluble in polar solvents, is a commercially available polymer with low cost. According to Fabiano et al., PEI can effectively dope n‐type polymers.^[^ ^19^ ^]^ We select PEI as the polymer dopant to blend with n‐PT5, which gives all polymer n‐type organic thermoelectric. Both PEI and n‐PT5 can be readily dissolved in HFIP. In the mixed solution of n‐PT5 (3 mg mL^−1^) and 6 wt% PEI in HFIP, the n‐PT5 polymer chains are molecularly dispersed in the alcohol solvent. This is proved by the dynamic light scattering results of the mixed solution (Figure S5, Supporting Information). Spin‐coating with the mixed solution affords the n‐PT5: PEI blend film.

**Figure 2:**
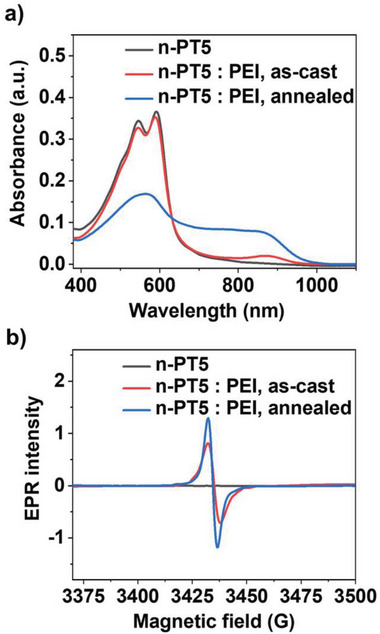
a) UV–vis–NIR absorption spectra of a thin film of n‐PT5 and 6 wt% PEI doped n‐PT5. b) Electron paramagnetic resonance (EPR) spectra of n‐PT5 and 6 wt% PEI doped n‐PT5 films.

The n‐doping of n‐PT5 by PEI is monitored by UV–vis–NIR absorption spectroscopy. The n‐PT5: PEI mixed solution shows a similar absorption spectrum to that of the n‐PT5 solution, indicating the absence of n‐doping in the solution (Figure S7, Supporting Information). In comparison, the as‐cast n‐PT5: PEI blend film exhibits a new absorption peak at ≈880 nm, which is attributed to the polaron of n‐PT5 and suggests the occurrence of n‐doping. After thermal annealing at 140 °C for 1.5 h, the n‐PT5: PEI blend film shows a greatly enhanced absorption band of n‐PT5 polaron and a significantly reduced absorption band of n‐PT5 itself, indicating the greatly increased doping level by thermal annealing. The n‐doping of n‐PT5 by PEI is confirmed by electron paramagnetic resonance (EPR) spectroscopy. As shown in **Figure** **2**b, while the pure n‐PT5 film does not produce EPR signals, the thermally annealed n‐PT5: PEI film generates robust spin signals. The spin density of the doped n‐PT5: PEI film was estimated to be 1.32 × 10^20^ cm^−3^ (see **Table** **1**) and the doping efficiency was calculated to be 15%.

**Table 1:** The optimal thermoelectric characteristics of PEI doped n‐PT5, *N*‐DMBI doped n‐PT5 films.

Polymer: Dopant	Optimal dopant concentration [wt%]	*σ*_max_ [S cm^−1^]	*S* ^a)^ [µV K^−1^]	*PF*_max_ ^b)^ [µW m^−1^ K^−2^]	Spin density [cm^−3^]	Doping efficiency [%]	Mobility [cm^2^ V^−1^ s^−1^]	Carrier concentration [cm^−3^]
n‐PT5: PEI	6	31.3	−108.6	36.9	1.32 × 10^20^	16.7	1.62 × 10^−2^	1.78 × 10^21^
n‐PT5: *N*‐DMBI	40	36.2	−69.0	18.2	1.45 × 10^20^	18.9	4.30 × 10^−3^	2.37 × 10^22^

Spin density and doping efficiency are obtained by electron paramagnetic resonance (EPR) measurements; Mobility and carrier concentration is obtained by the Hall‐effect measurements.

^a)^ Seebeck values at *PF*_max_;

^b)^ *PF* was calculated by multiplying the square of the mean of the Seebeck coefficient by the conductivity.

Both PEI and *N*‐DMBI can effectively n‐dope n‐PT5 in thin film after thermal annealing. The optimal n‐dopant concentration is 6 and 40 wt% for PEI and *N*‐DMBI, respectively. The Hall‐effect measurement (Table 1) was used to measure the charge carrier density and mobility of the n‐PT5: PEI and the n‐PT5: *N*‐DMBI films. Noteworthy, although charge mobilities in organic semiconductors determined by the Hall effect are often underestimated due to the non‐ideal Hall effect, these values based on the same polymer host are still of comparative significance.^[^ ^25^ ^]^ The carrier mobility of the n‐PT5: PEI film is 1.6 × 10^−2^ cm^2^ V^−1 ^s^−1^, which is much higher than that of the n‐PT5: *N*‐DMBI film (4.3 × 10^−3^ cm^2^ V^−1^ s^−1^). This result suggests that the polymer dopant is more effective than the small molecule dopant in terms of charging the host polymer while maintaining the charge transport pathway (Figure 4).

As n‐PT5 has polar OEG side chains and PEI is a polar polymer, in the n‐PT5: PEI blend film, the PEI polymer chains should be mainly distributed on the OEG side chain region and should be away from the n‐PT5 main backbone. Possible entanglement of PEI with the OEG side chains makes the micromorphology of n‐PT5: PEI blend more stable than the n‐PT5: *N*‐DMBI blend. Furthermore, due to the long OEG side chains, there is a large space between the stacked conjugated polymer chains, which can accommodate the polymer dopant PEI. This results in a lot of doping active sites on the tediously long PEI chains being far away from the conjugated polymer backbones in the n‐PT5: PEI blend film.

**Figure 3:**
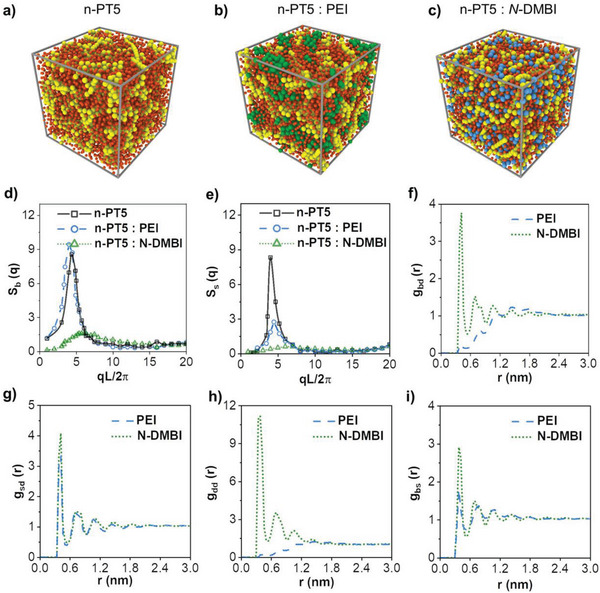
Typical MD simulation snapshots for microstructures of a) n‐PT5, b) PEI doped n‐PT5, and c) *N*‐DMBI doped n‐PT5, in which the yellow and red beads indicate the backbones and side chains of n‐PT5 polymers, the green beads denote the PEI and the blue beads represent the *N*‐DMBI. The size of side chain beads of n‐PT5 is reduced to highlight the morphologies of other species. Static structure factors of d) backbones S_b_(q) and e) side chains of n‐PT5 polymers with and without PEI or *N*‐DMBI dopant. Radial distribution functions of f) dopant (PEI or *N*‐DMBI) beads around each backbone bead of n‐PT5 [g_bd_(r)], g) dopant beads around each side chain bead of n‐PT5 [g_sd_(r)], h) dopant beads around each dopant bead [g_dd_(r)], and i) side chain beads around each backbone bead of n‐PT5 [g_bs_(r)].

**Figure 4:**
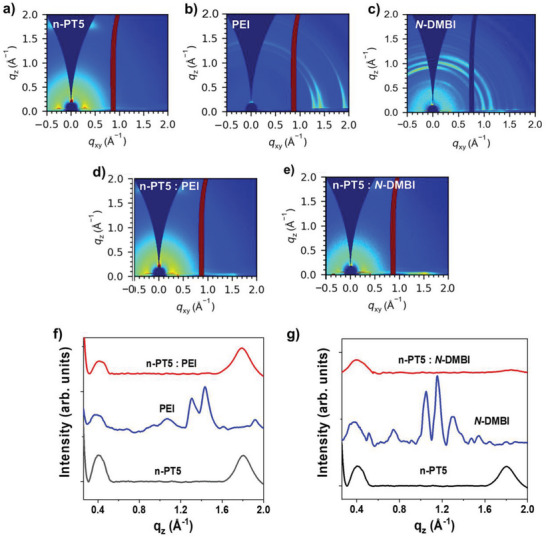
2D grazing incidence wide‐angle X‐ray scattering (GIWAXS) patterns of a) n‐PT5, b) PEI, c) *N*‐DMBI, d) PEI doped n‐PT5, e) *N*‐DMBI doped n‐PT5 films, f,g) The linecuts in the out‐of‐plane direction of the GIWAXS patterns. The measurement of the GIWAXS data was performed at the optimal concentration of the two dopants (6 wt% for PEI‐doped n‐PT5, and 40 wt% for N‐DMBI‐doped n‐PT5).

**Figure 5:**
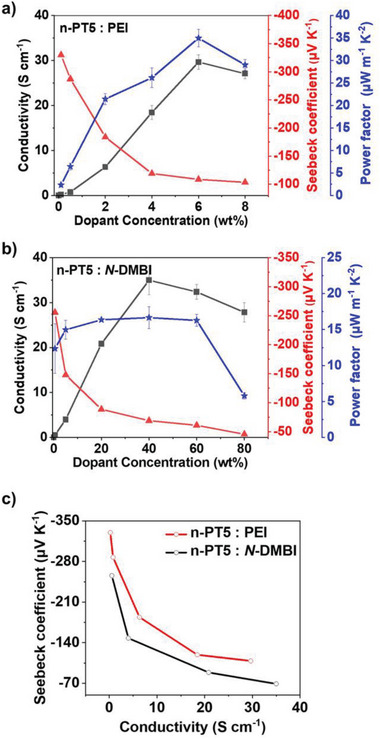
Dependence of electrical conductivities, Seebeck coefficients and power factors on dopant concentration of a) PEI doped n‐PT5 films, b) *N*‐DMBI doped n‐PT5 films. C) Experimental *S–σ* plots for PEI and *N*‐DMBI doped n‐PT5 films.

To fundamentally understand the microscopic mechanism for the thermoelectric performance in our work, we further employ coarse‐grained Molecular Dynamics (MD) simulations to study the micromorphology variations of n‐PT5 polymers caused by the doping of PEI or *N*‐DMBI. Herein, the model is developed based on our earlier simulation studies^[^ ^26^ ^]^ and other ref. [27] and the MD simulations are performed using the Large‐scale Atomic/Molecular Massively Parallel Simulator (LAMMPS).^[^ ^28^ ^]^ For clarity, the model (Figure S14, Supporting Information) and simulation details are briefly described in the Supporting Information. **Figure** **3**a–c shows the typical simulation snapshots for microstructures of n‐PT5 polymer, n‐PT5: PEI blend film, and n‐PT5: *N*‐DMBI blend film. We have calculated the static structure factor of the backbones (Sb(q)) and side chains (Ss(q)) of n‐PT5 with and without PEI or N‐DMBI. Additionally, we have determined the radial distribution functions of the dopants around the backbones [g_bd_(r)] and side chains [g_sd_(r)] of n‐PT5 polymer. We have also analyzed the dopant around the dopant [g_dd_(r)] and the side chains around the backbones of n‐PT5 polymer [g_bs_(r)] in Figure 3d‐i. Our simulations suggest that the unconjugated n‐PT5 polymer undergoes a typical microscopic phase separation where the n‐PT5 polymer backbones form bundle‐like structures through π–π interactions. Meanwhile, the hydrophilic oligo(ethylene glycol) side chains of the n‐PT5 polymer cluster in between these backbone bundles, as shown in the snapshots in Figure 3a. The apparent peaks of Sb(q) and Ss(q) at qL/2π≈5 in Figure 3d,e also support this observation. Additionally, we found that doping with *N*‐DMBI or PEI causes a slight change in the microstructures of the n‐PT5 polymer. Specifically, with the doping of *N*‐DMBI, the dopant shows affinities to both the backbones and side chains of the n‐PT5 polymer, which wrecks the original microscopic structures of the n‐PT5 polymer. In this context, there is a certain degree of aggregation of N‐DMBI, and it is more freely dispersed in the backbones and side chains, which causes the neighboring of dopant counterion and negatively charged polymer after the system ionization, as shown by the snapshot in Figure 3c and peaks of [g_bd_(r)], [g_sd_(r)], [g_dd_(r)] and [g_bs_(r)] at r <2 [nm] in Figure 3f‐i. Therefore, the n‐PT5: N‐DMBI blend film exhibits noncrystallinity and suboptimal thermoelectricity with a relatively low Seebeck coefficient (vide infra). In contrast, the polymer dopant is preferentially distributed in the side chain domains of n‐PT5, as shown by the snapshot in Figure 3b and the apparent peaks in [g_bd_(r)], [g_sd_(r)] and [g_dd_(r)] at r < 2.0 [nm] in Figure 3f–h. Meanwhile, the backbone aggregations of n‐PT5 are conserved, as shown by Sb(q) Figure 3d, which results in the unchanged crystallinity backbones as demonstrated by our experiments (**Figure** **4**). In addition, the PEI surrounded by the n‐PT5 side chains are alienated from the n‐PT5 backbones. Accordingly, after doping, considerable dislocations exist between the dopant counterion and negatively charged polymer, which reduces the Coulombic interaction and endows a relatively high Seebeck coefficient (**Figure** **5**). Our MD simulations thus confer the microstructures of n‐PT5 polymer film, n‐PT5: PEI blend film, and n‐PT5: N‐DMBI blend film on the molecular level and illustrate that the polymer dopants surrounded by the polymer side chains do not interfere with the ordered stacking of the polymer backbones. Considerable dislocations exist between the dopant counterion and negatively charged polymer after doping.

We used grazing incidence wide‐angle X‐ray scattering (GIWAXS)^[^ ^29^ ^]^ to determine the microstructure of the n‐PT5: PEI film. The results are shown in Figure 4. The n‐PT5 is primarily oriented face‐on on the substrate, exhibiting (010) diffraction peak at q_z_ = 1.80 Å^−1^ (d‐spacing = 3.50 Å) in the out‐of‐plane direction. In the n‐PT5: PEI blend film, the diffraction peaks of n‐PT5 are nearly unchanged and the diffraction peaks of PEI cannot be observed. This suggests that the ordered stacking of n‐PT5 conjugated polymer chains is well preserved and that the polymer dopant PEI is well dispersed in the n‐PT5 matrix. This is probably due to the excellent miscibility between the polar OEG side chains of n‐PT5 and the polar polymer dopant PEI. In comparison, for the n‐PT5: *N*‐DMBI blend, the (010) diffraction peak of n‐PT5 disappears, indicating that the ordered stacking of n‐PT5 conjugated polymer chains is broken and that the introduction of high content of small molecule dopants affects the stacking of polymer chains. Figure 7 and Figure S15 (Supporting Information) show the atomic force microscopy (AFM) images of the films of n‐PT5 itself, n‐PT5: PEI blend and n‐PT5: *N*‐DMBI blend. The surface of the n‐PT5: *N*‐DMBI blend is obviously rougher than those of the n‐PT5 itself and the n‐PT5: PEI blend. This is supported by the root‐mean‐square (RMS) roughness of 0.39, 0.42, and 0.92 nm for the n‐PT5 film, n‐PT5: PEI film, and n‐PT5: *N*‐DMBI film. These results imply that the the polymer dopant PEI is well dispersed in the n‐PT5 matrix and the small molecular dopant *N*‐DMBI forms aggregates in the n‐PT5 matrix in the blend films. Based on the GIWAXS and AFM results, we speculate that the doping of PEI mainly occurs in the amorphous region of the film and that the dopants are distributed primarily on the oligo(ethylene glycol) side chains region away from the backbone. After doping, there are considerable dislocations between the dopant counterion and negatively charged polymer. However, this does not interfere with the ordered stacking of the polymer skeleton. This agrees with the coarse‐grained MD simulation.

We assess the thermoelectric performance of n‐PT5 by measuring the electrical conductivity (σ) and Seebeck coefficient (S) of the n‐PT5: PEI blends and the n‐PT5: *N*‐DMBI blends in a nitrogen‐filled glovebox. Figure 5a displays the dependence of electrical conductivity on the dopant concentration of the two kinds of blends. The maximum *σ* values (σ_max_) are evaluated to be 31.3 S cm^−1^ for n‐PT5: PEI (dopant concentration: 6 wt%) and 36.2 S cm^−1^ for n‐PT5: *N*‐DMBI (dopant concentration: 40 wt%), respectively. As previously mentioned, PEI‐doped n‐PT5 exhibits relatively weaker charge/hydride transference. The comparable electrical conductivity of PEI and *N*‐DMBI doped n‐PT5 is attributed to the effective n‐doping and compact π‐stacking of polymer chains in the thin film after doping. The effective doping of conjugated polymers usually involves two processes:^[^ ^30^ ^]^ i) ionization of the dopant: n‐type dopant donates a charge or hydride to the host polymer and then forms Coulombically bound pairs between the ionized dopants and charged polymers; ii) carrierization: the free charge carriers generated from cation‐anion pairs. Previous studies have indicated that a large host‐dopant distance can help to reduce the charge localization by mitigating or screening the Coulombic interaction from counterions, thus enhancing the free charge generation. When n‐PT5 is doped with PEI, only 6% by weight of the dopant is needed to achieve the optimal conductivity of 31.3 S cm^−1^. In stark contrast, it takes 40% by weight of dopants for *N*‐DMBI doped n‐PT5 to achieve optimal conductivity. This could be due to the much higher equivalent weight of *N*‐DMBI compared to PEI. Another possible reason is the strong tendency of small molecules to aggregate out of the polymer matrics during the film‐forming process, which requires a high loading of small‐molecule dopants.

Negative Seebeck coefficients of the n‐PT5: PEI blends and the n‐PT5: *N*‐DMBI blends indicate electron‐dominant transport. It is interesting to note that the absolute Seebeck coefficient of the PEI‐doped n‐PT5 is much higher than that of the *N*‐DMBI‐doped n‐PT5, even at similar conductivities (Figure 5c). This could be due to the considerable distance between the dopant counterions and the charged polymer chains after doping, as supported by the MD simulation results. The increased host‐dopant distance reduces the Coulombic interaction between the dopant counterion and the charged polymer.^[^ ^22b^ ^]^ As the concentration of n‐doping increases, the Seebeck coefficient decreases because it negatively correlates with the carrier concentration. To determine the power factor (*PF*) as a function of doping, one can utilize the formula *PF* = *S^2^σ*. The highest power factor observed after doping n‐PT5 with PEI is 36.9 µW m^−1^ K^−2^, which is twice the power factor of *N*‐DMBI doped n‐PT5 (Table 1). The optimal values of conductivity and power factor are achieved simultaneously in n‐PT5 doped with 6 wt% PEI. Figure 5c displays the *S*‐*σ* plots of PEI‐doped and *N*‐DMBI‐doped n‐PT5 films. It seems that the *S*‐*σ* curve of the former is upright lifted as compared to the latter. This indicates that the enhanced power factor of the PEI‐doped n‐PT5 is not due to the change in the doping levels. The underlying reason is possibly the increased host‐dopant distance, which has been elaborated by a previous study.^[^ ^22b^ ^]^

**Figure 6:**
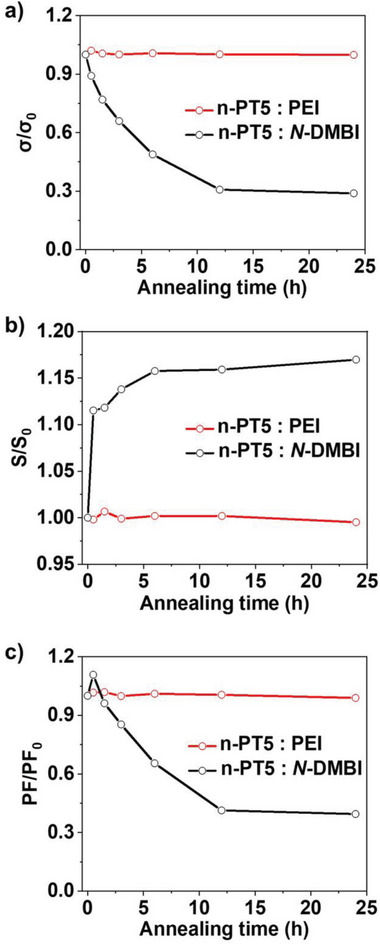
a) Normalized electrical conductivity, b) Normalized Seebeck coefficients, c) Normalized power factor of PEI doped n‐PT5, *N*‐DMBI doped n‐PT5 films on continuous thermal annealing at 140 °C for 24 h inside a N_2_‐filled glovebox.

As thermoelectric materials need to work continuously under thermal stress, excellent thermal stability is essential. We compare the thermal stability of n‐PT5: PEI to that of n‐PT5: *N*‐DMBI. The thermoelectric performance of the n‐PT5: PEI blend film shows negligible attenuation after thermal annealing at 140 °C for 24 h under an inert atmosphere (**Figure** **6**). In contrast, the electrical conductivity and power factor of the n‐PT5: *N*‐DMBI blend film decrease by over 70% and 60% respectively, upon annealing at 140 °C for 24 h. Our results prove that the thermal stability of n‐PT5: PEI blend film is far superior to n‐PT5: *N*‐DMBI blend film.

**Figure 7:**
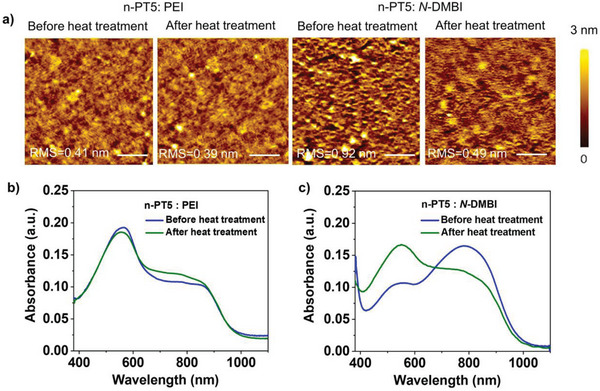
a) AFM height images of the thin film of PEI doped n‐PT5, *N*‐DMBI doped n‐PT5 before and after continuous heat treatment at 140 ^ o^C for 24 h inside an N_2_‐filled glovebox. The root‐mean‐square (RMS) surface roughness is provided in the height images. The scale size is 2 µm × 2 µm (Scale bar: 500 nm). UV–vis–NIR absorption spectra of b) PEI doped n‐PT5 films, c) *N*‐DMBI doped n‐PT5 films before and after heat treatment 24 h.

**Figure** **7** displays the AFM height of n‐PT5: PEI and n‐PT5: *N*‐DMBI films before and after the thermal annealing. After the thermal annealing, while the RMS roughness of the n‐PT5: PEI film surface does not change obviously, the RMS roughness of the n‐PT5: *N*‐DMBI film surface decreases significantly from 0.92 to 0.49 nm. This suggests the excellent stability of the polymer host /polymer dopant blend and the limited morphological stability of the polymer host /small molecule dopant blend. Small molecule dopants, which have poor thermal stability, tend to sublime or diffuse out of doped films during thermal annealing more easily than polymer dopants. This is evident from the mass loss observed by TGA (Figure S4, Supporting Information). We also compare the UV–vis absorption spectra of the two blend films before and after thermal annealing (Figure 7b,c). For the n‐PT5: *N*‐DMBI film, the polaron absorption intensity obviously decreased and the absorption intensity of n‐PT5 itself obviously increased, suggesting de‐doping after the thermal annealing. In comparison, the n‐PT5: PEI film does not show obvious change after the thermal annealing, indicating negligible de‐doping after thermal annealing. The UV–vis–NIR absorption spectroscopy results are in accordance with the AFM results and confirm the stable doping of all polymer n‐type thermoelectrics.

## 3 Conclusion

We have developed an alcohol‐soluble n‐type conjugated polymer, n‐PT5, by attaching polar and branched OEG side chains to the cyano‐substituted poly(thiophene‐alt‐co‐thiazole) main chain. High‐performance n‐type all‐polymer thermoelectrics are demonstrated by blending n‐PT5 with a low content of polymer dopant PEI. Compared to the polymer host/small molecule dopant blends, the polymer host/polymer dopant blends have the advantages of enhanced Seebeck coefficient and consequently higher power factor as well as improved thermal stability. This is probably due to the reduced Coulombic interaction between the dopant counterions and the charged polymer main chains, which leads to well‐preserved ordered stacking of the polymer host. Our work provides a novel method for improving the Seebeck coefficient at a given conductivity and offers feasible guidelines for optimizing the doping behavior toward high‐performance n‐type organic thermoelectrics.

## Conflict of Interest

The authors declare no conflict of interest.

## Supporting information

Supporting Information

## Data Availability

The data that support the findings of this study are available in the supplementary material of this article.
