# Supplementary material for: Alcohol‐Processable All‐Polymer n‐Type Thermoelectrics
Source: Adv Sci (Weinh). 2024 Apr 22;11(25):2401952. doi: 10.1002/advs.202401952 (PMC11220645; doi:10.1002/advs.202401952)
Supplement: Supplementary file 1 — Supporting Information [file ADVS-11-2401952-s001.pdf]

ADVANCED SCIENCE
Open Access

Supporting Information

for *Adv. Sci.*, DOI 10.1002/advs.202401952

Alcohol-Processable All-Polymer n-Type Thermoelectrics

Xinyi Fan, Jian Liu, Xiaozheng Duan, Hongxiang Li, Sihui Deng, Yazhuo Kuang, Jingyu Li, Chengjiang Lin, Bin Meng, Junli Hu*, Shumeng Wang*, Jun Liu* and Lixiang Wang*

Supporting Information

Alcohol-Processable All-Polymer n-Type Thermoelectrics

Xinyi Fan, Jian Liu, Xiaozheng Duan, Hongxiang Li, Sihui Deng, Yazhuo Kuang, Jingyu Li, Chengjiang Lin, Bin Meng, Junli Hu,* Shumeng Wang,* Jun Liu,* and Lixiang Wang*

Table of Contents

1. Characterization
2. DFT calculations
3. Materials and synthetic procedures
4. Molecular weights and molecular weight distribution
5. Thermal properties
6. Dynamic light scattering (DLS)
7. Cyclic voltammetry (CV)
8. UV-vis-NIR absorption spectra
9. Electron paramagnetic resonance (EPR) spectra
10. Hall-effect measurements
11. Thermoelectric properties
12. Coarse-grained Molecular Dynamics (MD) simulation
13. Atomic force microscope (AFM)
14. ^{1}H NMR and ^{13}C NMR spectra
15. References

1. Characterization

General. ^{1}H NMR and ^{13}C NMR spectra of organic molecules were measured with a Bruker AV-500 spectrometer in $CDCl_3$, C_6D_6 or $(CD_3)_2SO$ at 25 °C. ^{1}H NMR spectra of polymers were recorded on a Bruker AV-400 in $C_6D_4Cl_2$ at 110 °C. Elemental analysis was performed on a VarioEL elemental analyzer. Gel permeation chromatography (GPC) was carried out at 25 °C with a PL-220-type GPC. Hexafluoroisopropanol (HFIP) was used as the eluent and mono-disperse polystyrene was used as the reference. Thermal gravimetric analysis (TGA) was performed under an N_2 flow at a heating rate of 10 °C/min with a Perkin-Elmer-TGA 7 system. The absorption spectra were measured using a PerkinElmer UV/VIS spectrometer Lambda 35 with a home-built closed quartz colorimeter for protecting the samples from the air exposure during absorption measurement. The thickness of pristine or doped films was measured with a Bruker 3D Optical Profilometer Contour GT-I. Atomic force microscopy (AFM) was performed with a SPA300HV (Seiko Instruments, Inc., Japan) in tapping mode. Electron paramagnetic resonance (EPR) spectra were recorded with a Bruker EMXnano EPR spectrometer running the Xenon software at room temperature (The microwave power was 0.3162 mW; the modulation amplitude was 4.000 G; the modulation frequency was 100.00 kHz; the time constant was 1.28 ms; and the sweep time was 100.00 s). Samples were made by adding the polymer/blend solution to a quartz tube and rapidly removing the solvent to mimic the spin coating behavior. GIWAXS data were obtained at 1W1A Diffuse X-ray Scattering Station, Beijing Synchrotron Radiation Facility (BSRF-1W1A). The monochromatic of the light source was 1.54 Å. The data were recorded by using the two-dimensional image plate detector of Eiger 2M from Dectris, Switzerland. The film used in 2D-GIWAXS tests was prepared by spin-casting (3 mg/mL). The configuration optimizations of the polymer were conducted by Density functional theory (DFT) calculations using the Gaussian 09 program at the B3LYP/6-31G (d,p) level of theory on the neutral model molecules of each polymer. Long oligo(ethylene glycol) side chains were replaced with methyl groups to simplify calculation. The dynamic light scattering tests were performed at 25 °C using a Zetasizer Nano ZS device equipped with a He-Ne laser (X=633 nm). A total of three parallel tests were conducted.

2. DFT calculations

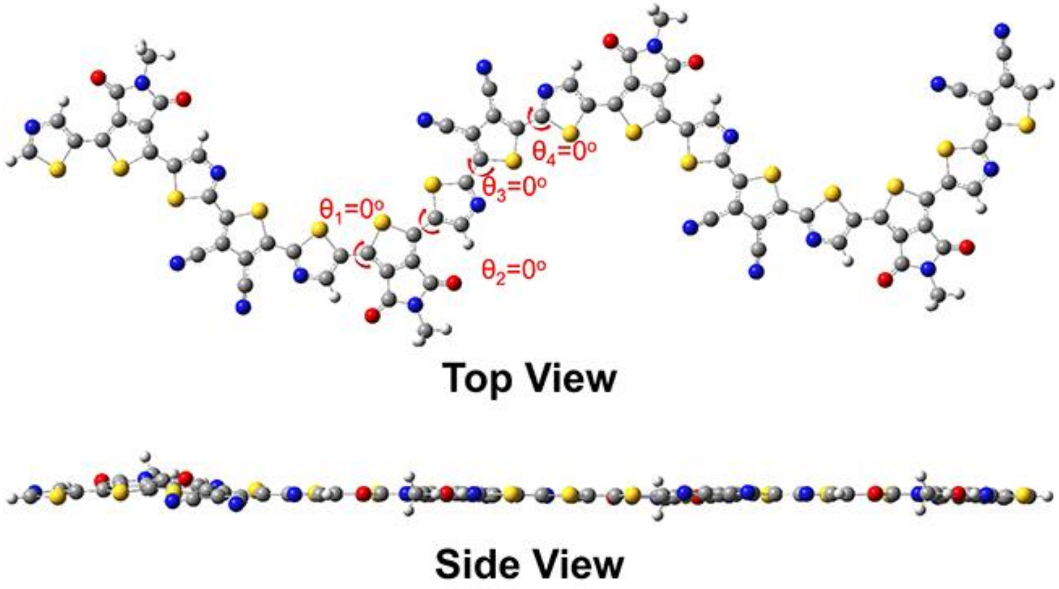

Figure S1. DFT-optimized geometries of the tetramers of the polymer backbone of n-PT5. Long oligo(ethylene glycol) side chains were replaced with methyl groups to simplify calculation.

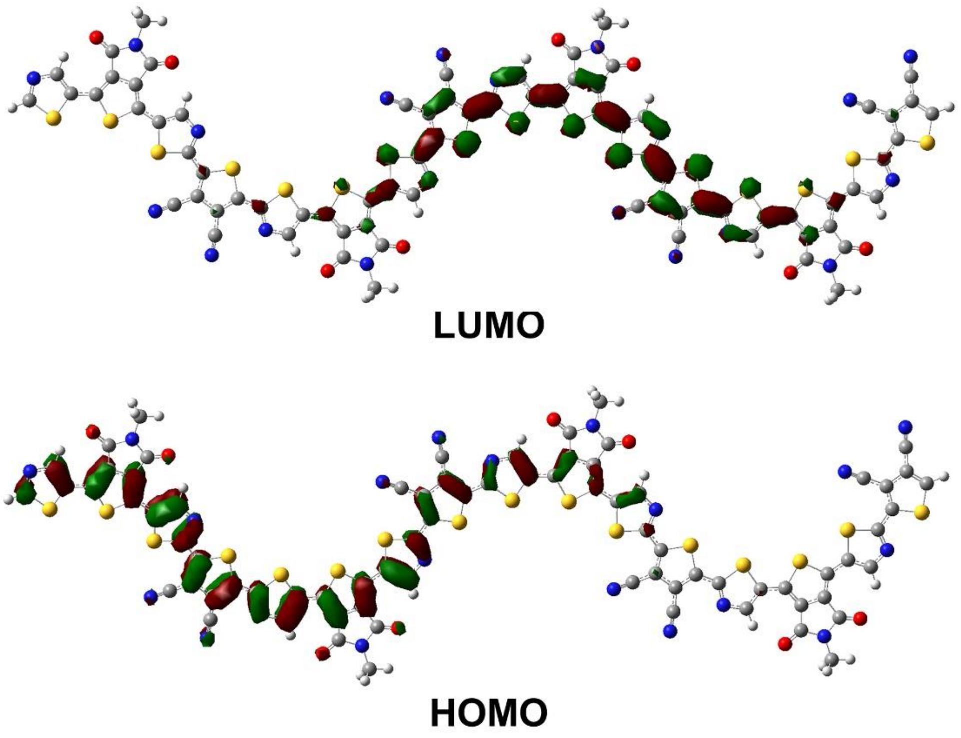

Figure S2. Calculated Kohn-Sham LUMOs/HOMOs based on four repeating units of n-PT5. Long oligo(ethylene glycol) side chains were replaced with methyl groups to simplify calculation.

3. Materials and synthetic procedures

Materials. All reactions were performed under argon atmosphere, unless stated otherwise. Commercially available solvents and reagents were used without further purification unless otherwise mentioned. Dry ethyl ether, toluene and tetrahydrofuran were distilled via standard methods. Ultra-dry dimethyl formamide were obtained from Adamas-beta®. The dopants PEI and N-DMBI used in this work were purchased from Beyotime Biotechnology and Macklin, respectively. PEI has a molecular weight of 25000 and a degree of polymerization of 580.

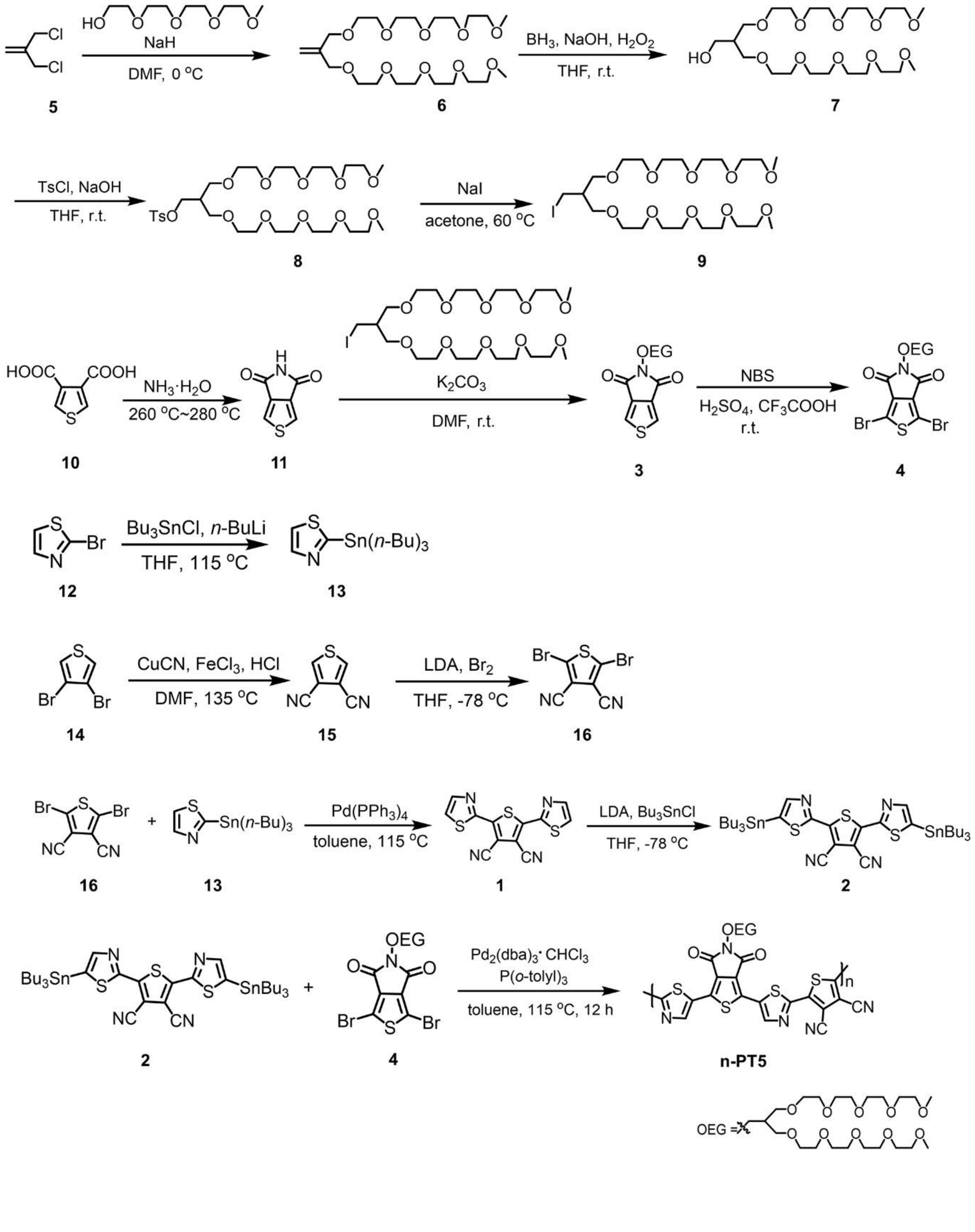

Scheme S1. Detailed synthetic routes of n-PT5.

Synthesis of 13: 12 (4.50 g, 27.4 mmol) was dissolved in ethyl ether (120 mL) in a flask. The solution of *n*-BuLi in *n*-hexane (12.1 mL, 30.2 mmol) was then slowly added to the flask at -78 °C. After stirring at -78 °C for 1.5 hours, Bu_3SnCl (10.3 g, 31.6 mmol) was slowly added,

stirring at -78 ^{o}C for another 1 hour. The reaction system was restored to room temperature and diluted with ethyl ether (100 mL). The crude product was washed three times with distilled water. The combined organic layers were dried over anhydrous Na_2SO_4, concentrated with a rotary evaporator to afford **13** as a colorless oil (10.1 g, yield: 98%).

Synthesis of 1: The synthesis of **16** is according to the previously reported methods.[1] A mixture of **16** (0.35 g, 1.20 mmol), **13** (1.35 g, 3.61 mmol), Pd $(PPh_3)_4$ (0.14 g, 0.12 mmol) and degassed toluene (36 mL) was stirred at 115 ^{o}C under argon atmosphere for 22 hours. After removal of the solvent, the crude product was purified by column chromatography using dichloromethane/ethyl acetate (60:1) as the eluent to a give the pure product **1** as a white solid (0.25 g, Yield: 69%).^{1}H NMR (500 MHz, $CDCl_3$) δ = 8.01 (d, 2H), 7.66 (d, 2H).

Synthesis of 2: Fresh made 1.0 M Lithium diisopropylamide (3 mL, 3 mmol) in tetrahydrofuran was added dropwise at -78 ^{o}C to a solution of **1** (0.30 g, 1.00 mmol) in anhydrous tetrahydrofuran (60 mL). After stirring for 3.5 hours at -78 °C, Bu_3SnCl (1.04 g, 3.20 mmol) was added in one portion to the reaction mixture. Stirring at -78 ^{o}C overnight. After removal of the solvent, the crude product was purified by column chromatography using hexanes/dichloromethane (3:5) as the eluent to a give the pure product **2** as a colorless oil (0.68 g, Yield: 77%). ^{1}H NMR (500 MHz, C_6D_6) δ = 7.92 (s, 2H), 1.51 (m, 12H), 1.29 (m, 12H), 1.04 (m, 12H), 0.89 (m, 18H). ^{13}C NMR (126 MHz, C_6D_6) δ = 160.88, 150.43, 146.59, 133.86, 112.37, 108.83, 28.85, 27.09, 13.43, 10.96.

Synthesis of 9: The synthesis of **8** is according to the previously reported methods.[2] A mixture of 7 (3.71 g, 5.79 mmol), NaI (1.74 g, 11.61 mmol) and degassed acetone (15 mL) was stirred at 60 ^{o}C under argon atmosphere for 18 hours. The solution was poured into 30 mL of water. The mixture was extracted with dichloromethane. The combined organic layers were dried over anhydrous Na_2SO_4, concentrated and the residue was purified by silica gel chromatography (hexanes/ethyl acetate/methanol = 2:1:0.3) to afford **9** as a colorless oil (3.38 g, yield: 98%). ^{1}H NMR (500 MHz, $CDCl_3$) δ = 3.65 (m, 36H), 3.38 (s, 6H), 3.36 (d, 2H), 1.93 (m, 1H).

Synthesis of 3: The synthesis of **11** is according to the previously reported methods.[3] **11** (0.77 g, 5.03 mmol), K_2CO_3 (1.39 g, 10.06 mmol) was added to a flask, then 50 mL of dimethyl formamide was added slowly. Then slowly add **9** to the flask, stirring at room temperature overnight. The solution was poured into water (50 mL). The mixture was extracted with

dichloromethane. The combined organic layers were dried over anhydrous Na_2SO_4, concentrated and the residue was purified by silica gel chromatography (hexanes/ethyl acetate/methanol = 2:1:0.5) to afford **3** as a colorless oil (2.55 g, yield: 82%). ^{1}H NMR (500 MHz, $CDCl_3$) δ = 7.81 (s, 2H), 3.72 (d, 2H), 3.65 (m, 36H), 3.38 (s, 6H), 2.41 (m, 1H).

Synthesis of 4: N-bromosuccinimide (0.55 g, 3.09 mmol) was added to a solution of **3** (0.51 g, 0.97 mmol) in the mixture of trifluoroacetic acid (12 mL) and sulfuric acid (4 mL). The solution was stirred for 8 h in the dark at room temperature. Then the solution was poured into 50 mL cold water. The mixture was extracted with dichloromethane. The combined organic layers were dried over anhydrous Na_2SO_4, concentrated and the residue was purified by silica gel chromatography (hexanes/ethyl acetate/methanol = 2:1:0.3) to afford **4** as a colorless oil (0.82 g, yield: 85%).^{1}H NMR (500 MHz, $CDCl_3$) δ = 3.69 (d, 2H), 3.66 (m, 37H), 3.38 (s, dH), 2.38 (m, 1H).

Synthesis of n-PT5: A mixture of **2** (180.1 mg, 0.205 mmol), **4** (156.7 mg, 0.201 mmol), $Pd_2(dba)_3{\cdot}CHCl_3$ (4.2 mg, 0.0033 mmol), P(*o*-tolyl)$_3$ (9.8 mg, 0.026 mmol) and degassed toluene (7 mL) was vigorously stirred at 115 ^{o}C under argon atmosphere for 12 hours. After cooling down, the resulting mixture was poured into methanol (150 mL) and the precipitate was collected by filtration. The crude polymer was washed in a Soxhlet apparatus first with acetone and then with n-hexane and finally with chloroform. The chloroform fraction was concentrated and poured into methanol. The polymer was recovered by filtration and dried in vacuum overnight (137.0 mg, Yield = 74%). ^{1}H NMR (400 MHz, C_6D_6) δ = 8.60 (s, 2H), 3.56 (m, 42H), 3.25 (m, 6H), 2.53 (m, 2H). Anal. Calcd for $C_{40}H_{49}N_5O_{12}S_4$: C, 52.22; H, 5.37; N, 7.60. Found: C, 51.77; H, 5.17; N, 7.53. GPC (HFIP, polystyrene standard, 25 °C): Mn = 98.9 kDa, PDI = 1.92.

4. Molecular weight and molecular weight distribution

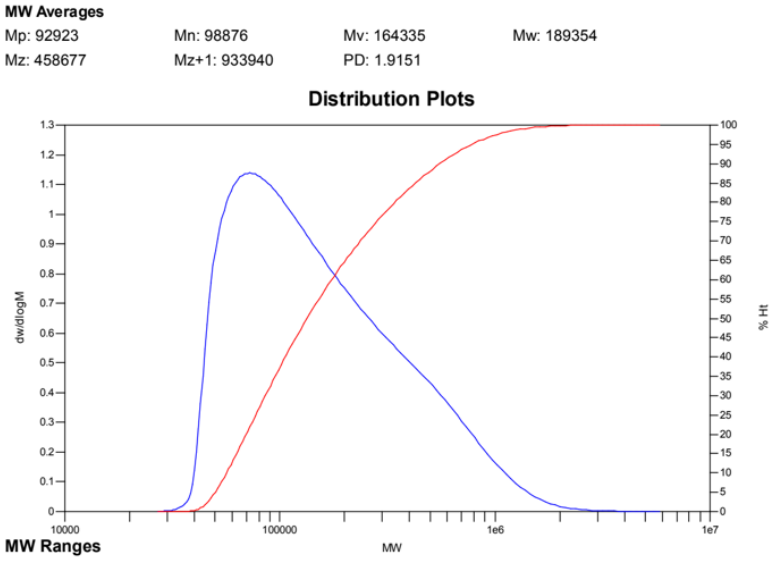

Figure S3. Gel permeation chromatography (GPC) elution curves of n-PT5 with HFIP as the eluent at 25 °C.

5. Thermal properties

The thermal properties of the n-PT5, PEI and *N*-DMBI were determined by thermal gravimetric analysis (TGA) under N_2 flow. n-PT5 shows good thermal stability with thermal decomposition temperatures (T_d) at 5% weight loss of 340 °C. PEI and *N*-DMBI shows the T_d at 5% weight loss of 296 °C and 186 °C, respectively.

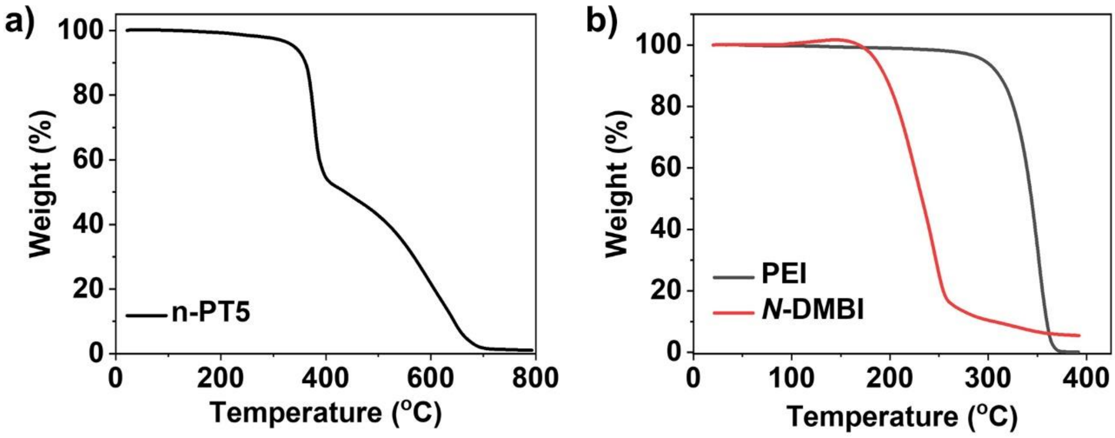

Figure S4. Thermogravimetric analysis (TGA) curves of a) n-PT5 and b) dopants.

6. Dynamic light scattering (DLS)

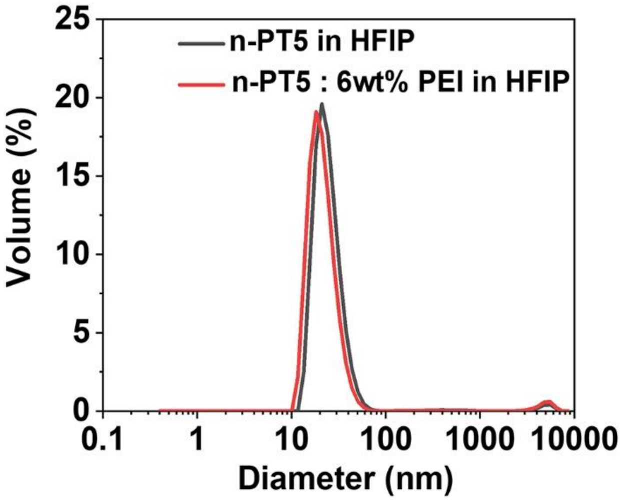

Figure S5. Hydrodynamic diameter of n-PT5 and n-PT5: PEI blend solution in HFIP determined by dynamic light scattering.

7. Cyclic voltammetry (CV)

Cyclic voltammetry (CV) was performed in a solution of 0.1 M NH_4PF_6 in acetonitrile with a CHI660a electrochemical analyzer system using a glassy carbon working electrode, a platinum disk counter electrode and a saturated calomel reference electrode. The polymer materials were cast on the working electrode for measurement. The redox potentials were calibrated with ferrocene as an internal standard. The highest occupied molecular orbital (HOMO) and the lowest unoccupied molecular orbital (LUMO) energy levels of the materials were estimated by the equations: HOMO/LUMO = – (4.80 + E_{onset}^{ox}/E_{onset}^{red}). The reduction curves of n-PT5 were scanned in multiple turns using cyclic voltammetry. We also used ferrocene/ferrocenium as the internal standard for CV measurement, and the difference in results for LUMO levels is less than 0.02 eV.

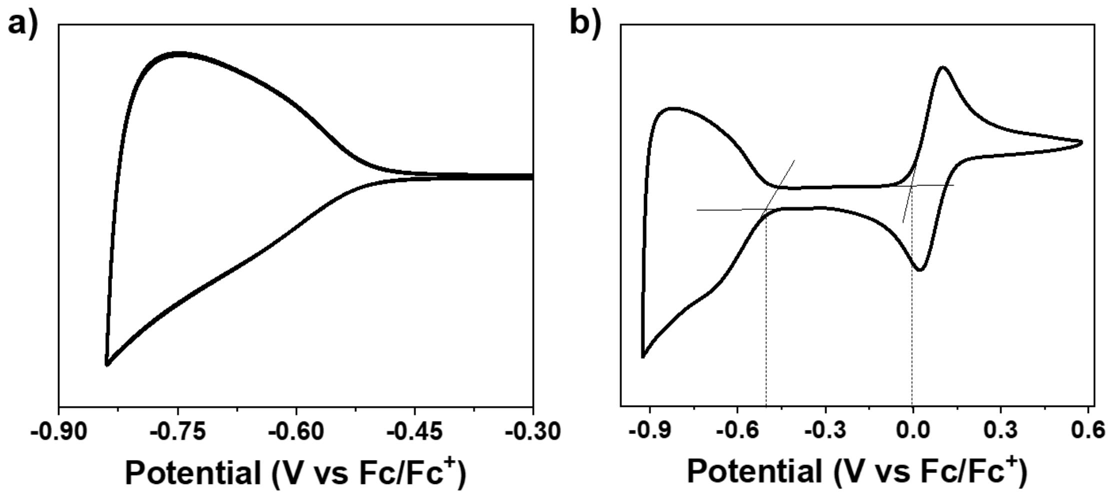

Figure S6. Cyclic voltammograms of n-PT5 in thin film of a) the reduction curves scanned in multiple turns (the three circles basically coincide) and b) ferrocene/ferrocenium as the internal standard.

8. UV-vis-NIR absorption spectra

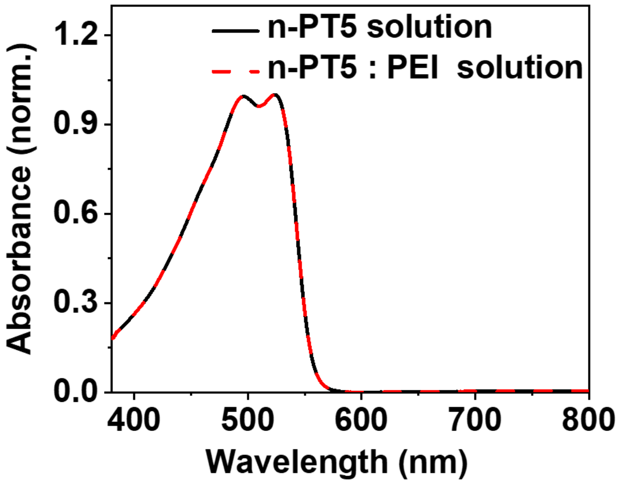

Figure S7. UV-vis-NIR absorption spectra of n-PT5: PEI blend solution.

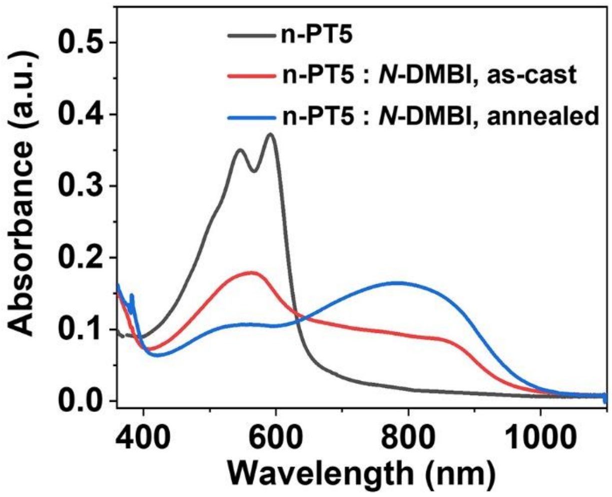

Figure S8. UV-vis-NIR absorption spectra of n-PT5 and *N*-DMBI doped n-PT5 films.

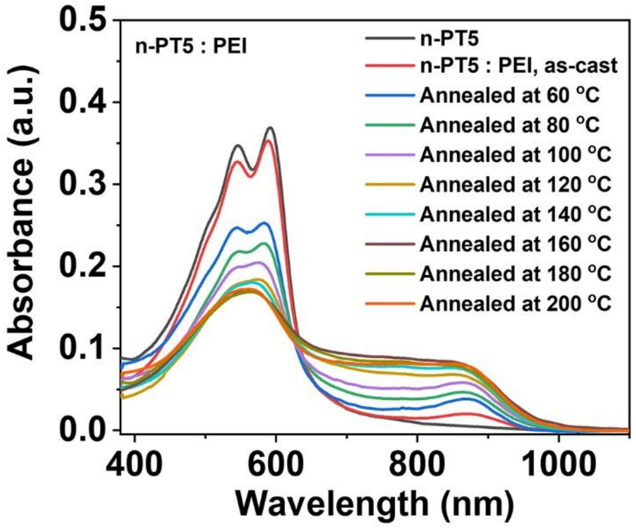

Figure S9. UV-vis-NIR absorption spectra of PEI doped n-PT5 films annealed at different temperatures for 1.5 hours. Pristine n-PT5 film was tested as a control.

9. Electron paramagnetic resonance (EPR) spectra

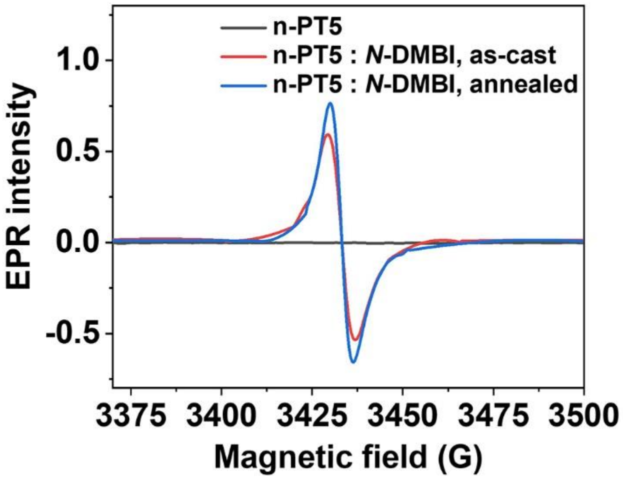

Figure S10. Electron paramagnetic resonance (EPR) spectra of the *N*-DMBI doped n-PT5 films. Pristine n-PT5 film was tested as a control.

10. Hall-effect measurements

Hall measurements were performed by a Lakeshore M91 FastHall™ measurement controller with Fast Hall mode at a field amplitude of 890 mT. The Hall measurement devices were fabricated and encapsulated as follows. Firstly, the 17×17 mm^2 glass substrates were deposited of Cr+Au (2+40 nm) electrodes, as shown in Figure S10. The polymer solutions were spin-coated onto the patterned electrodes and annealed at 150 ^oC 30 min. When the devices were cooled to room temperature, excess films were wiped off with a cotton swab dipped in ethanol to enclose the doped film to the tips of electrodes. The devices were encapsulated as a "sandwich structure" by another 15×15 mm^2 glass substrate using UV epoxy as an adhesive to block the doped film from air.

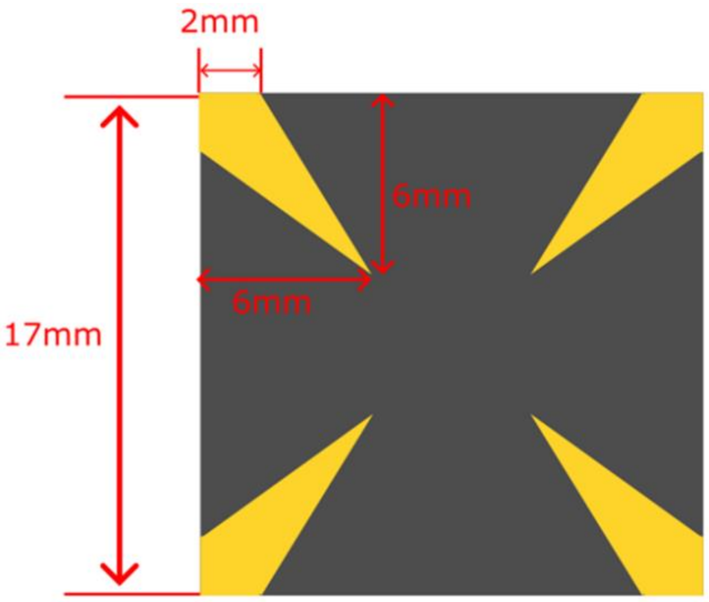

Figure S11. Schematic view of electrode template for M91 Fast Hall measurement.

11. Thermoelectric properties

Device fabrication. The borosilicate glass was sequentially cleaned using ultrasonication in deionized water (twice), acetone (twice), and isopropanol (once), 10 min for each time. The substrates were heated at 120 ^{o}C for 30 min and finally treated through UV-zone instrument for 15 min. The undoped polymer films were then spin-coated onto the substrate from HFIP solution (3 mg/mL) in a glovebox under nitrogen atmosphere. The doped films were prepared by spin-coating the conjugated polymer solution (3 mg/mL in HFIP for n-PT5) mixed with different amounts of dopant solution (3 mg/mL in HFIP) on glass substrates patterned with electrodes in a glovebox with nitrogen atmosphere. The resultant films were annealed at 140 ^{o}C for 1.5 hours. The vacuum-deposited electrodes, with a channel length of 200 μm and a channel width of 1000 μm for conductivity measurements and a channel length of 5000 μm and a channel width of 4000 μm for Seebeck coefficient measurements.

Electrical conductivity measurements. The measurements of thermoelectric properties are similar to our previous study [3-4]. The electrical conductivity (σ) was measured with probe station and Keysight B1500A under argon atmosphere. Conductivity (σ) calculated by using the equation: $\sigma=(I/V)\times L/(w\times d)$, where I is the current, V is the voltage, d is the thickness of the

film, L and w are the length and width of the channel. The conductivities of four separate points were averaged to obtain the conductivity of one device.

Seebeck coefficient measurements. The Seebeck coefficient was measured under argon atmosphere and calculated by the formula: $S = V_{\text{therm}}/\Delta T$, where V_{therm} is the thermovoltage obtained by creating the temperature difference (ΔT) at the two ends of the channel. The V_{therm} was tested by Keithley 2000, and the temperature difference was created by two Peliter elements and tested by an infrared camera FLIR A300 (thermal sensitivity < 50 mK). To calibrate our setup, commercial constantan wire (127 μm from Omega) and high purity nickel wire were also measured.[5] The Seebeck coefficient measurement by using our home-built setup is quite reliable.

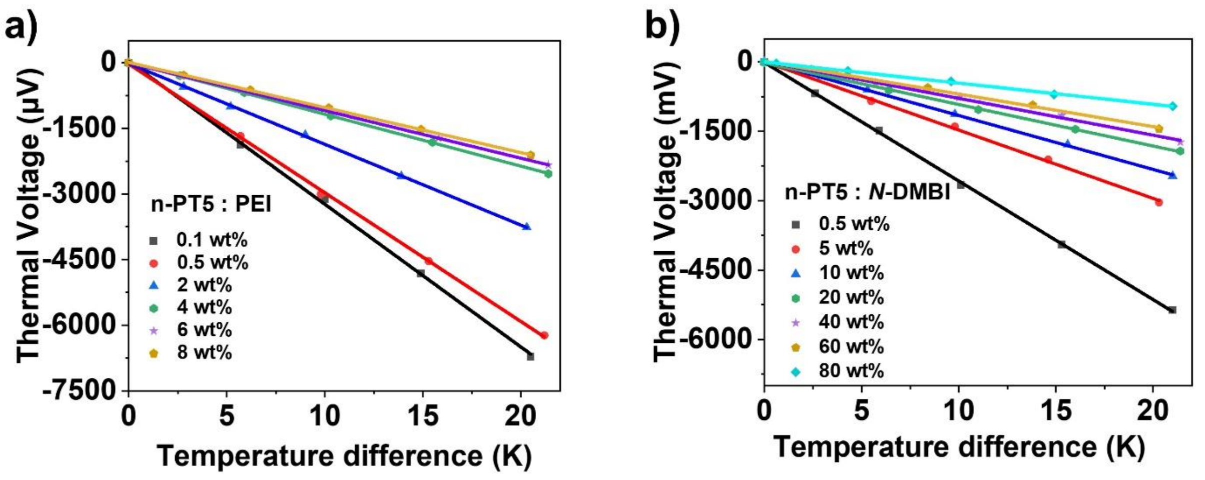

Figure S12. Temperature difference dependent thermal voltage of a) PEI and b) *N*-DMBI doped n-PT5 films at different dopant concentration.

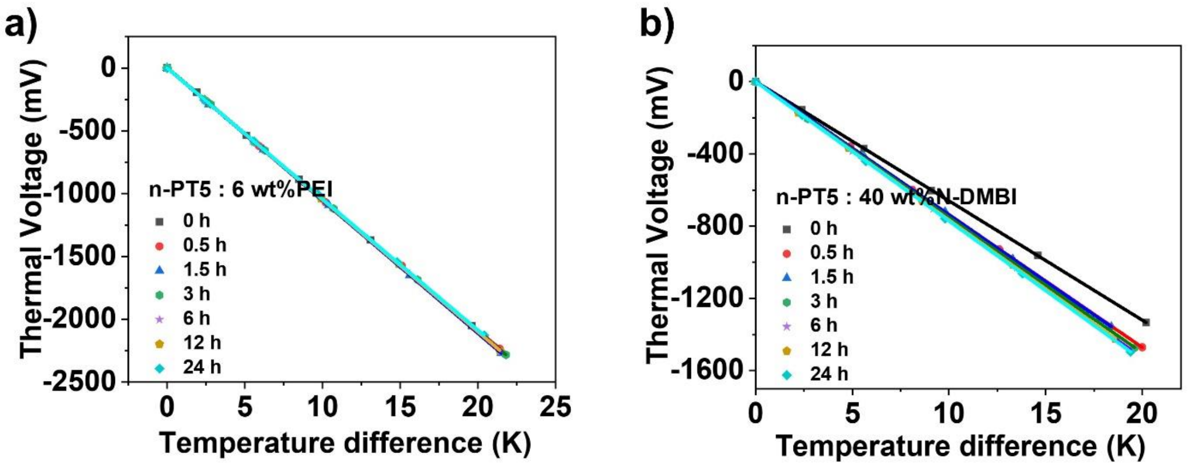

Figure S13. Temperature difference dependent thermal voltage of a) 6 wt% PEI and b) 40 wt% *N*-DMBI doped n-PT5 films after heat treatment of different time.

12. Coarse-grained Molecular Dynamics (MD) simulation

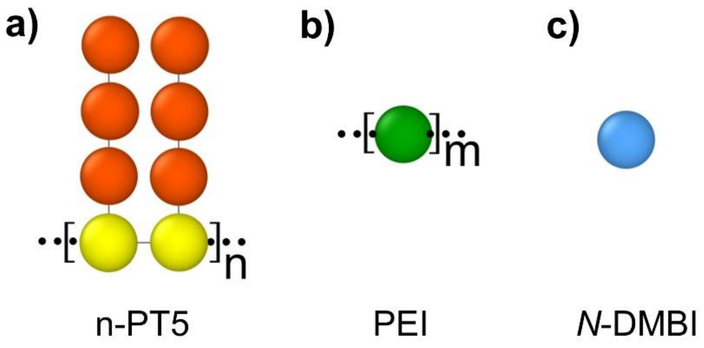

Figure S14. Coarse-grained model of a) a grafted n-PT5 polymer chain composed of a backbone (in yellow) and side chains (in red), b) a linear PEI polymer chain and c) an *N*-DMBI molecule.

We use the coarse-grained Molecular Dynamics (MD) simulation to study the microstructural variations of the n-PT5 polymers caused by the hybridization of *N*-DMBI molecules and PEI polymers. Herein, the model is constructed based on our previous simulations[6] and other Ref [7]. We perform the simulation in the canonical (NVT) ensemble in a cubic box using the Large-scale Atomic/Molecular Massively Parallel Simulator (LAMMPS) package[8].

In our simulation, we coarse-grain each n-PT5 polymer as a grafted chain consisting of a backbone and side chains composed of spherical beads (Figure S14 a), and model each PEI polymer as a linear freely-jointed chain composed of spherical beads (Figure S14 b). The polymerization degrees of n-PT5 and PEI are set as $n = 60$ and $m = 77$, respectively. In addition, we model the *N*-DMBI molecules as independent spherical beads (Figure S14 c). For simplicity, we assume that all the aforementioned beads have the same diameters $\sigma = 1.0$ (corresponding to 4 Å in the real unit), and these beads are further categorized into nonpolar, polar and charged beads in accordance with the specific chemical structures of n-PT5, PEI and *N*-DMBI molecules[7]. We account for the *non-bonded* interactions between the beads using the truncated and shifted Lennard-Jones (LJ) potential,

$$U_{LJ}(r) = \begin{cases} 4\varepsilon_{\mathrm{LJ}}\left[\left(\frac{\sigma}{r}\right)^{12} - \left(\frac{\sigma}{r}\right)^{6} + S\right], r \le r_c \\ 0, r > r_c \end{cases} \qquad \text{Equation S1}$$

where the energetic parameter is varied as $\varepsilon_{\mathrm{LJ}} = 1.0 \sim 1.5$ (which corresponds to $1.0 \sim 1.5\ k_{\mathrm{B}}T$ at T = 298 K) and the cutoff is adjusted from $r_{\mathrm{c}} = 2^{1/6}\sigma$ to $r_{\mathrm{c}} = 2.5\sigma$ to model the molecular interactions, including the volume excluded effect, π-π interactions between n-PT5 backbones, as well as hydrophobic/hydrophilic interactions and solvation interactions. In addition, σ = 1.0 (which corresponds to 4 Å) and $\varepsilon_{\mathrm{LJ}}^0$ = 1.0 (which corresponds to $k_{\mathrm{B}}T$ at room temperature) are set as the length and energy units, and the LJ potential is shifted to 0 at r_c by S. We model the bonding effects between the conjunctive beads in the n-PT5 and PEI polymers through the finitely extensible nonlinear elastic (FENE) potential,

$$U_{\mathrm{FENE}}(r_b) = -\frac{1}{2}kR_0^2 \ln\left[1-\left(\frac{r_b}{R_0}\right)^2\right] \qquad \text{Equation S2}$$

where $k = 30\varepsilon_{\mathrm{LJ}}^0/\sigma^2$ is the spring constant and $R_0 = 1.5\ \sigma$ represents the maximum bond extension. We employ the harmonic angular potential to calculate the intrinsic rigidity of the n-PT5 backbones,

$$U_{\mathrm{Angle}}(\theta) = \kappa_{\mathrm{a}}\left(\theta - \theta_0\right)^2 \qquad \text{Equation S3}$$

where θ is the angle of between conjective beads (i-1, i and i +1), θ_0 is set as 180° and harmonic parameter is fixed as κ_{a} = 10. Further, due to the anionization of n-PT5 and cationization of PEI or N-DMBI, we use Coulomb potential to account for the electrostatic interactions between the ionized beads,

$$U_{\mathrm{Coul}} = \frac{Z_1 Z_2 e^2}{4\pi\varepsilon_0\varepsilon_{\mathrm{r}} r} \qquad \text{Equation S4}$$

in which Z_1 and Z_2 are the valences of the ionized beads 1 and 2 with a separation distance r, ε_0 indicates the vacuum permittivity and the dielectric constant of the polymer mixtures is set as ε_{r} = 20. Here, U_{Coul} is calculated using the PPPM (particle-particle-particle-mesh) algorithm[9] with an accuracy of 10^{-5}.

In all these simulations, the three-dimensional periodic boundary conditions are applied. The integration MD time step is set as 0.005 (which corresponds to 9 fs) and the temperature is controlled via the Langevin thermostat. To study the hybridization of *N*-DMBI and PEI on the structural variations of n-PT5 polymers, we consider the following representative cases: i) for the case without hybridization, 19 n-PT5 polymers are randomly generated in the simulation box; ii) for the n-PT5: *N*-DMBI mixture, 16 n-PT5 polymers and 1315 PEI are randomly

generated in the simulation box; and iii) for the n-PT5 : PEI mixture, 16 n-PT5 polymers and 16 PEI are randomly generated in the simulation box. For these three cases, we adjust the size of the simulation box, so that the total number density of the beads is set as ρ ~ 0.85. Note that the molar ratio between n-PT5 and *N*-DMBI and between n-PT5 and PEI are comparable to our experimental data. In the simulations, we first use 10^6 MD steps simulation at T_0 = 413 K to obtain the equilibrated structures of the complexes only through Equation S1-S3, after which we perform another 10^6 MD steps simulation as the cooling procedure until the temperature decreases to $T_1 = 0.1T_0$. We then account for the ionization of the system: for the n-PT5 polymers without hybridization [case i)], the system is maintained as uncharged; for the n-PT5 : *N*-DMBI mixture [case ii)], we randomly set 20% n-PT5 beads as negatively charged and 20% *N*-DMBI beads as positively charged; and for the n-PT5 : PEI mixture [case iii)], we randomly set 16% n-PT5 beads as negatively charged and 16% PEI beads as positively charged. Note that the ionization degrees in case ii) and case iii) are consistent with the cases in our experiments. We turn on the electrostatic interactions and perform 10^6 MD steps at $T_1 = 0.1T_0$ to achieve the new equilibration of the system, and further use another 10^6 MD steps as the production runs. For all these three cases of n-PT5 polymers, n-PT5: *N*-DMBI mixture and n-PT5: PEI mixture, we perform 20 simulations with different initial system configurations and obtain the final results through statistical samplings.

13. Atomic force microscope (AFM)

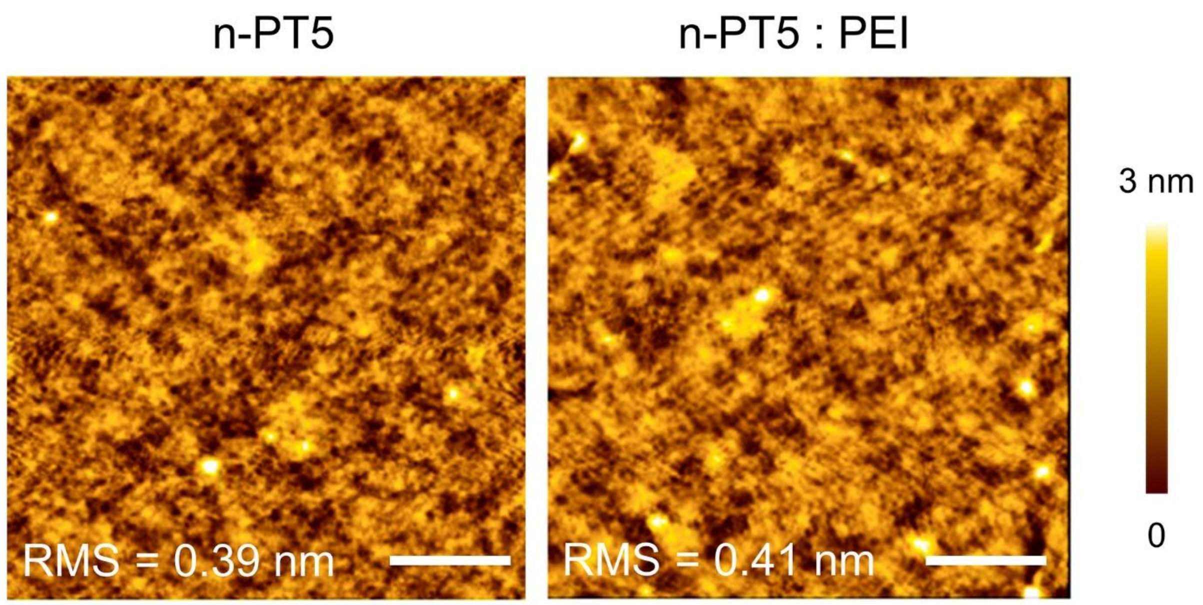

Figure S15. AFM height images of n-PT5 and PEI doped n-PT5 films. Scale bars: 500 nm.

14. ¹H NMR and ¹³C NMR spectra

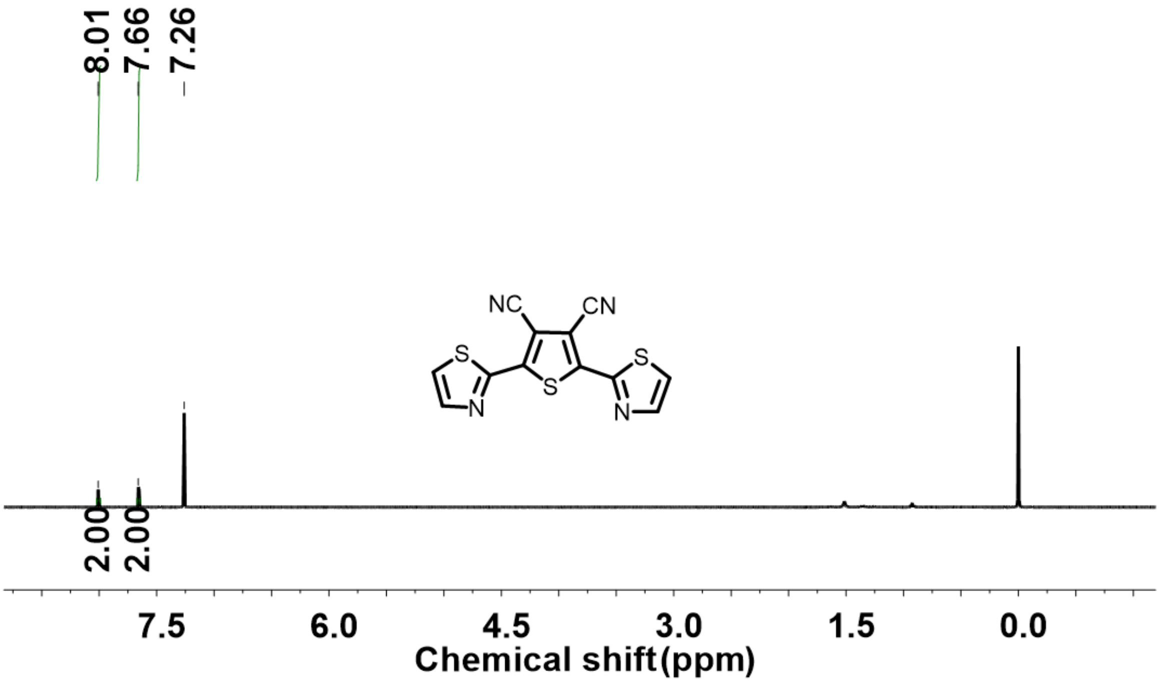

¹H NMR spectrum of **1** in $CDCl_3$

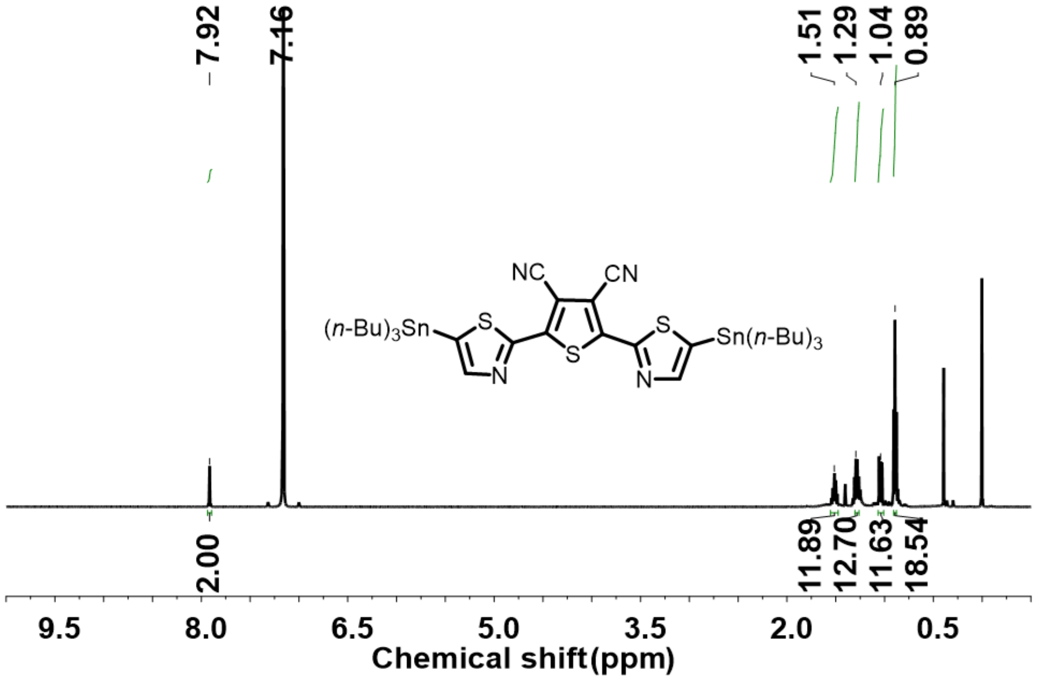

^{1}H NMR spectrum of 2 in C_6D_6

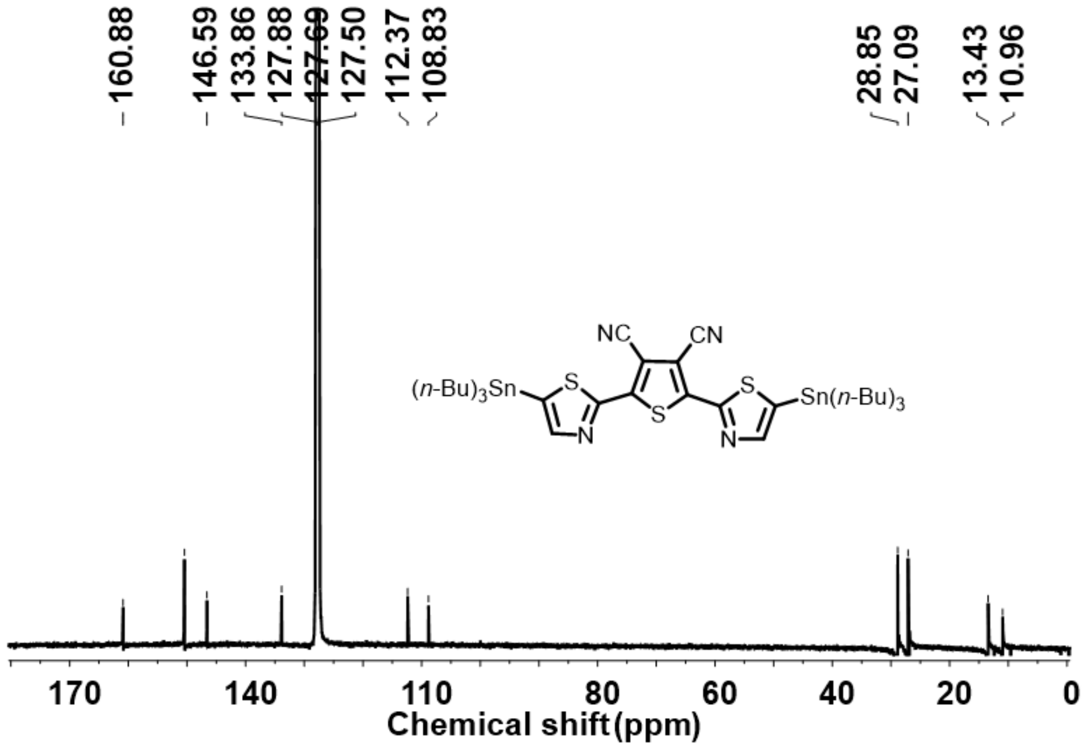

^{13}C NMR spectrum of 2 in C_6D_6

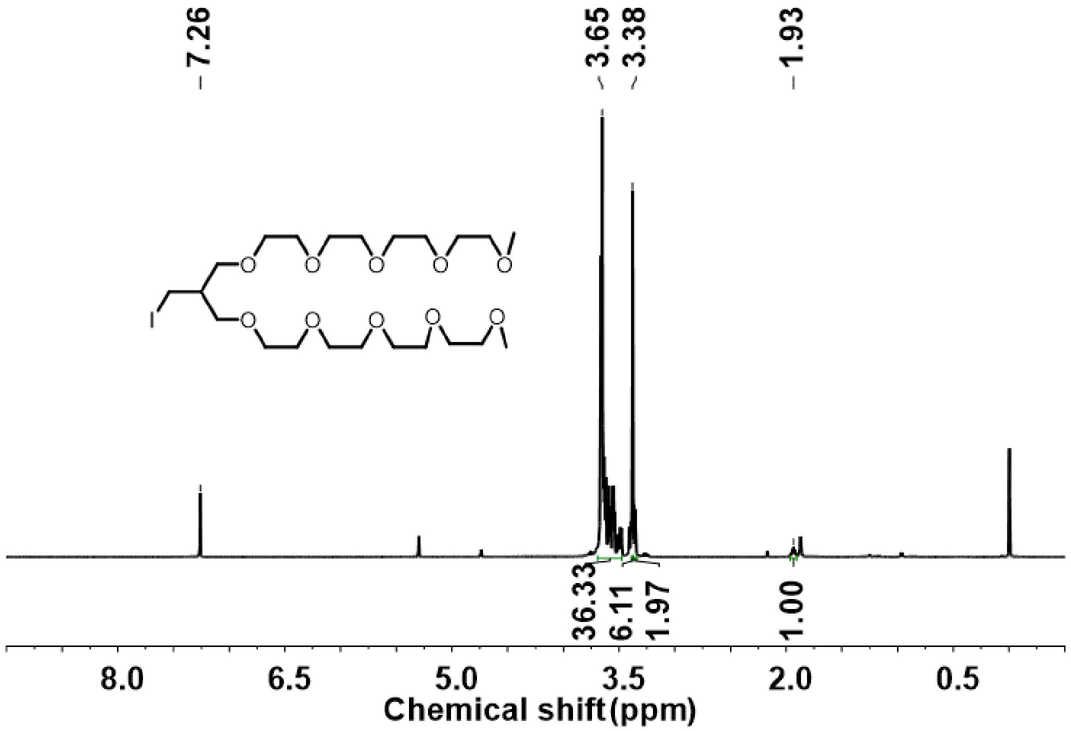

^{1}H NMR spectrum of 9 in $CDCl_3$

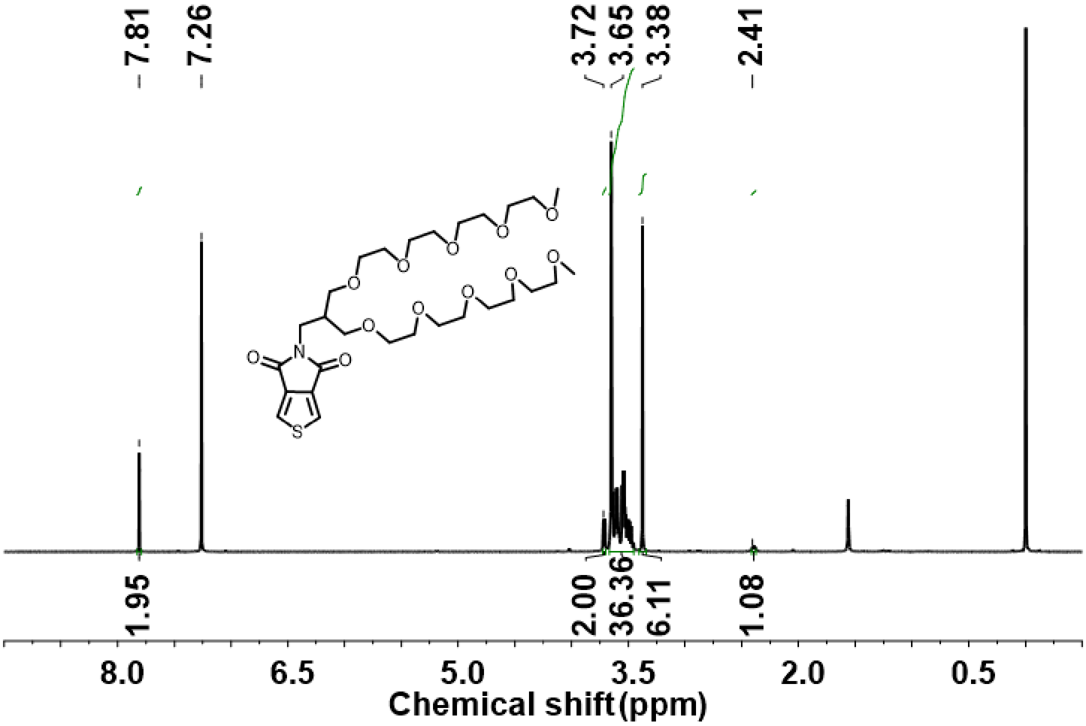

^{1}H NMR spectrum of 3 in $CDCl_3$

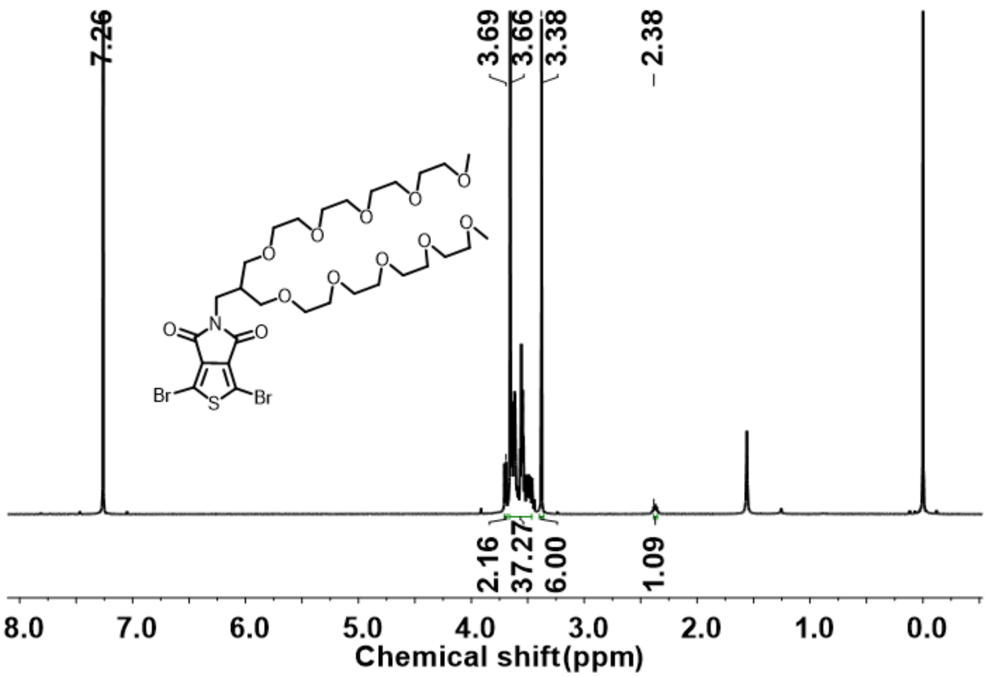

^{1}H NMR spectrum of 4 in $CDCl_3$

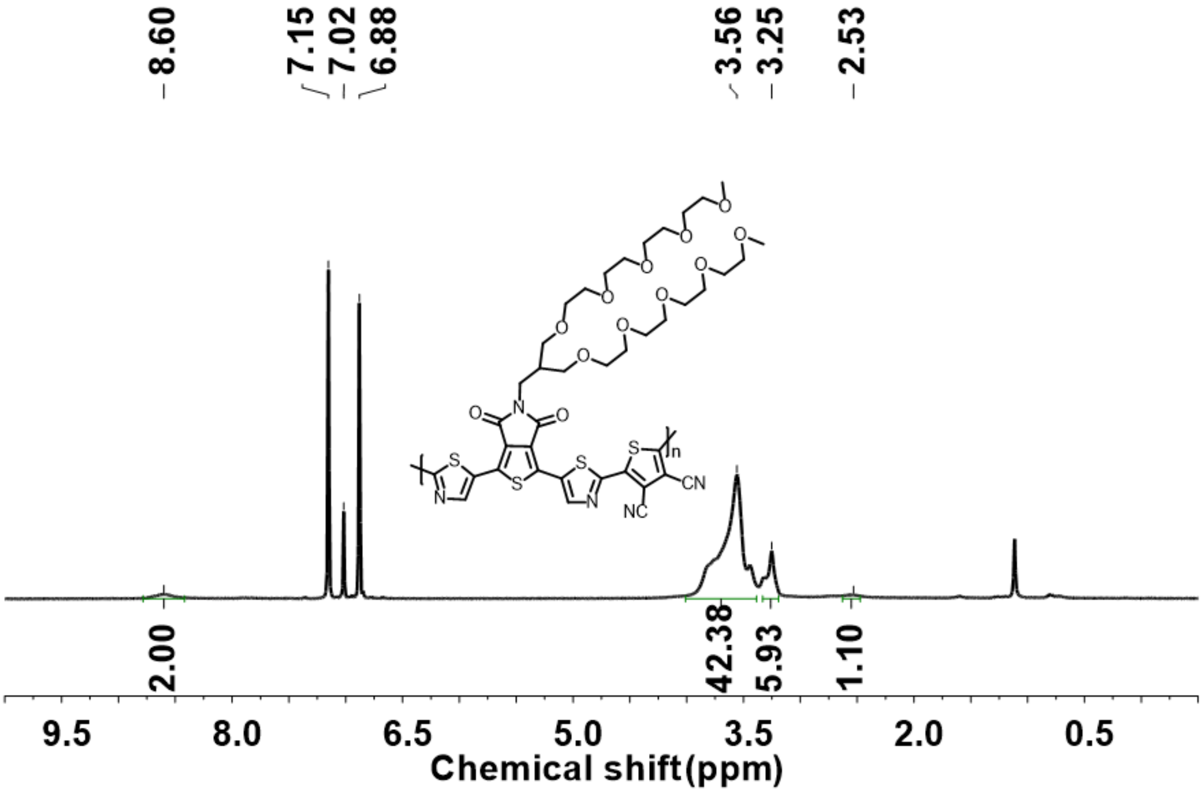

^{1}H NMR spectrum of n-PT5 in C_6D_4Cl

References

[1] J. Y. Balandier, F. Quist, C. Amato, S. Bouzakraoui, J. Cornil, S. Sergeyev, Y. Geerts, *Tetrahedron* **2010**, *66*, 9560-9572.

[2] M. Lee, Y. S. Jeong, B. K. Cho, N. K. Oh, W. C. Zin, C*hem. Eur. J.* **2002**, *8*, 876-883.

[3] S. H. Deng, Y. Z. Kuang, L. Y. Liu, X. Y. Liu, J. Liu, J. Y. Li, B. Meng, C. Di, J. L. Hu, J. Liu, *Adv. Mater.* **2023**, DOI: 10.1002/adma.202309679.

[4] a) S. Deng, C. Dong, J. Liu, B. Meng, J. Hu, Y. Min, H. Tian, J. Liu, L. Wang, *Angew. Chem. Int. Ed.* **2023**, *62*, *e202216049*; b) C. Dong, S. Deng, B. Meng, J. Liu, L. Wang, *Angew. Chem. Int. Ed.* **2021**, *60*, 16184-16190.

[5] C. S. Dong, B. Meng, J. Liu, L. X. Wang, *ACS Appl. Mater. Interfaces* **2020**, *12*, 10428-10433.

[6] a) Z.-D. Wang, S. Liang, Y. Yang, Z.-N. Liu, X. Duan, X. Li, T. Liu, H.-Y. Zang, *Nat. Comm.* **2023**, *14*, 2767; b) B. Li, X. Duan, D. Cheng, X. Chen, Z. Gao, W. Ren, K.-Z. Shao, H.-Y. Zang, *J. Am. Chem. Soc.* **2022,** *144*, 6434-6441; c) Z. Zhu, X. Duan, Q. Li, R. Wu, Y. Wang, B. Li, *J. Am. Chem. Soc.* **2020**, *142*, 4481-4492; d) L. Chao, Y. Xia, X. Duan, Y. Wang, C. Ran, T. Niu, L. Gu, D. Li, J. Hu, X. Gao, J. Zhang, Y. Chen, *Joule* **2022**, *6*, 2203-2217; e) B. Liu, B. Hu, J. Du, D. Cheng, H. Y. Zang, X. Ge, H. Tan, Y. Wang, X. Duan, Z. Jin, *Angew. Chem. Int. Ed.* **2021**, *133*, 6141-6150; f) Y. Zhang, C. Yi, W. Dong, D. Zheng, Y. Yang, W. Li, X. Duan, D. Yang, Z. Nie, *Adv. Funct. Mater.* **2022**, *32*, 2112742; g) X. Duan, A.-C. Shi, L. An, *Macromolecules* **2021**, *54*, 9053-9062; h) H. Huo, W. Zhao, X. Duan, Z.-Y. Sun, *Macromolecules* **2023**, *56*, 1065-1076; i) C. Lin, H. Wei, H. Li, X. Duan, *Soft Matter* **2022**, *18*, 1603-1616; j) J. Liang, H. Wei, K. Yu, C. Lin, H. Li, M. Ding, X. Duan, *Soft Matter* **2021**, *17*, 6305-6314.

[7] S. J. Marrink, H. J. Risselada, S. Yefimov, T. D. Peter, A. H. de Vries, *J. Phys. Chem. B* **2007**, *111*, 7812-7824.

[8] S. Plimpton, *J. Comp. Phys.* **1995**, *117*, 1-19.

[9] T. Darden, D. York, L. Pedersen, *J.Chem. Phys.* **1993**, *98*, 10089-10092.
